# DARPP-32 interaction with adducin may mediate rapid environmental effects on striatal neurons

**DOI:** 10.1038/ncomms10099

**Published:** 2015-12-07

**Authors:** Olivia Engmann, Albert Giralt, Nicolas Gervasi, Lucile Marion-Poll, Laila Gasmi, Odile Filhol, Marina R. Picciotto, Diana Gilligan, Paul Greengard, Angus C. Nairn, Denis Hervé, Jean-Antoine Girault

**Affiliations:** 1 Inserm UMR-S 839, Paris 75005, France; 2 Sorbonne Universités, UPMC, Université Paris 06, Paris 75005, France; 3 Institut du Fer à Moulin, 17 rue du Fer à Moulin, Paris 75005, France; 4 Inserm, U1036, CEA, 17 rue des Martyrs, Grenoble 38054, France; 5 Department of Psychiatry, Yale University School of Medicine, 300 George Street, Suite 901, New Haven, Connecticut 06511, USA; 6 Upstate University Hospital, SUNY Upstate University, 5309 Weiskotten Hall, 766 Irving Avenue, Syracuse, New York 13210, USA; 7 Laboratory of Molecular and Cellular Neuroscience, The Rockefeller University, 1230 York Avenue, New York, New York 10065, USA

## Abstract

Environmental enrichment has multiple effects on behaviour, including modification of responses to psychostimulant drugs mediated by striatal neurons. However, the underlying molecular and cellular mechanisms are not known. Here we show that DARPP-32, a hub signalling protein in striatal neurons, interacts with adducins, which are cytoskeletal proteins that cap actin filaments’ fast-growing ends and regulate synaptic stability. DARPP-32 binds to adducin MARCKS domain and this interaction is modulated by DARPP-32 Ser97 phosphorylation. Phospho-Thr75-DARPP-32 facilitates β-adducin Ser713 phosphorylation through inhibition of a cAMP-dependent protein kinase/phosphatase-2A cascade. Caffeine or 24-h exposure to a novel enriched environment increases adducin phosphorylation in WT, but not T75A mutant mice. This cascade is implicated in the effects of brief exposure to novel enriched environment on dendritic spines in nucleus accumbens and cocaine locomotor response. Our results suggest a molecular pathway by which environmental changes may rapidly alter responsiveness of striatal neurons involved in the reward system.

## Abstract (web summary)

Changes in environment are known to alter reward system responses, although the underlying mechanisms are unclear. Here, Engmann *et al.* show that DARPP-32 interacts directly with β-adducin in the mouse striatum to regulate structural and behavioural plasticity in response to novel environment and drug exposure.

## Graphical abstract

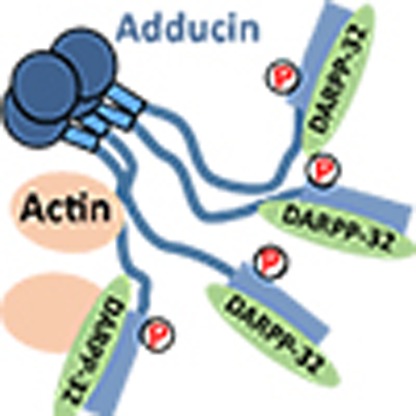

Brain reward systems play a central role in adaptive behaviours and have been highly conserved during evolution. The diversion of these systems by addictive substances or behaviours has profound consequences on human comportment, society and health, with a huge economic impact^1^. Diverse addictive stimuli share the ability to enhance dopamine signalling and modulate reward-related learning and memory^2^,^3^; yet, the responsiveness of humans and animal models to drugs is highly dependent on a variety of genetic and environmental factors that are not fully understood. For example, only a fraction of individuals exposed to cocaine ultimately become addicted^4^. Moreover, enriched environments alter behavioural effects of addictive drugs^5^, although little is known about the molecular mechanisms of such modulation of reward responses.

The medium-sized spiny neurons of the striatum are major components of reward and motor systems and primary targets of dopamine innervation. These neurons express high levels of specific signalling proteins, including DARPP-32 (32-kDa dopamine- and cAMP-regulated phosphoprotein, protein phosphatase-1 regulatory subunit 1B (PPP1R1B)), a hub for several signalling pathways regulated by multiple extracellular signals^6^,^7^. DARPP-32 functions as a switch, reinforcing or inhibiting the action of the cAMP-dependent pathway, depending on its state of phosphorylation. When phosphorylated by cAMP-dependent protein kinase (PKA) on Thr34, DARPP-32 inhibits protein phosphatase-1, participating in an open positive feed-forward loop^8^. In contrast, when DARPP-32 is phosphorylated by Cdk5 on Thr75 it is an inhibitor of PKA^9^. DARPP-32 is involved in acute and long-term responses of medium-sized spiny neurons at multiple levels, from synapses to nucleus^10^. The regulation of DARPP-32 phosphorylation is well characterized but its protein partners are not known, beyond the enzymes it regulates or targets.

We report here that β-adducin is a major interaction partner of DARPP-32. Adducins are actin-capping proteins that stabilize the cortical cytoskeleton^11^,^12^. β-adducin regulates dendritic spine stability^13^,^14^. It is thought to play a role in learning and memory^13^,^14^,^15^,^16^ through its action in actin-based synapse formation and spectrin-based synapse stabilization^13^,^17^, which is essential in response to enriched environment^13^. Cocaine can induce phosphorylation of β-adducin by protein kinase C (PKC)^18^ and mice lacking β-adducin show impairments in fear conditioning and spatial learning, and increased cocaine sensitization^14^,^15^,^16^.

In the current study we describe β-adducin interaction with DARPP-32 and dissect how this interaction affects phosphorylation of the two proteins. We show that when DARPP-32 is phosphorylated on Thr75 it enhances β-adducin phosphorylation on Ser713. We report the existence of alterations of dendritic spines in the nucleus accumbens and cocaine locomotor effects as early as 24 h after exposure of mice to a novel enriched environment (NEE). These effects are prevented by DARPP-32 Thr75 point mutation or in the absence of β-adducin, suggesting a role of these proteins in the rapid neuronal modifications induced by environment.

## Results

### DARPP-32 binds to the β-adducin MARCKS domain

**Figure 1:**
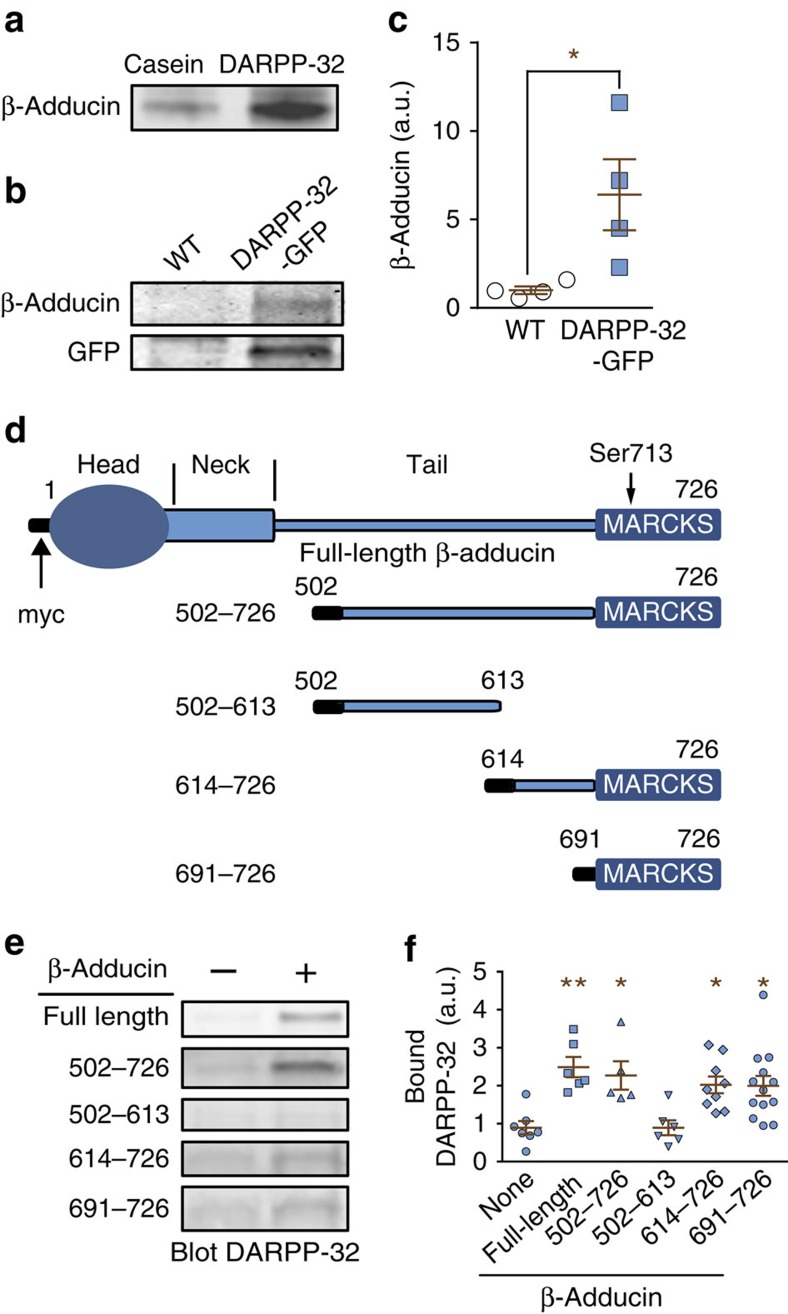
DARPP-32 interacts with the MARCKS domain of β-adducin. (**a**) Rat striatal lysates were incubated with Sepharose coupled to purified recombinant DARPP-32 or to casein as control. After washing, proteins were eluted with 150 mM NaCl, analysed by SDS–polyacrylamide gel electrophoresis and immunoblotted for β-adducin. (**b**) Striatal lysates from DARPP-32-GFP transgenic mice and WT littermates were incubated with Sepharose-bound GFP antibody. Immune precipitates were analysed by immunoblotting for β-adducin (upper panel) or GFP (lower panel). (**c**) The amounts of β-adducin co-immunoprecipitated with GFP from WT or DARPP-32-GFP transgenic mice as in **c** were quantified. Independent data points are plotted and means±s.e.m. are shown, *n*=4 per group, Student’s *t*-test, *t*_6_=2.68, *P<0.05. (**d**) Domain organization of β-adducin and its fragments used in the current study (all fused to an N-terminal 13-residue myc peptide, black). MARCKS, myristoylated alanine-rich C-kinase substrate homology domain. The position of the phosphorylated Ser713 in the MARCKS domain is indicated. (**e**) COS-7 cells were transfected with empty myc vector (−) or myc-tagged full-length β-adducin or its fragments (+). Myc-tagged proteins were immunoprecipitated with agarose-coupled myc antibody. Beads were then incubated with lysates of DARPP-32-GFP-transfected cells and bound DARPP-32 detected by immunoblotting. (**f**) Quantification of DARPP-32-GFP association with β-adducin fragments. The background level is indicated by the amount of DARPP-32 precipitated with beads coated with lysates of myc vector-transfected COS-7 cells (−, dotted line). Independent data points are plotted and means±s.e.m. are shown; *n*=5–13 per group from >3 experiments; one-way ANOVA: *F*_(5,40)_=5.82, *P*<0.001. Tukey‘s test versus no β-adducin (-): *P<0.05; **P<0.01. Independent data points are plotted and means±s.e.m. are shown.

To identify DARPP-32 interacting partners, we immobilized purified rat DARPP-32 to a Sepharose matrix. Casein, a protein comparable to DARPP-32 in size and acidity, was used as negative control. Sepharose beads were incubated with precleared rat striatal lysate and eluted with a salt gradient. Eluted proteins were separated by polyacrylamide gel electrophoresis and excised bands were analysed by liquid chromatography–mass spectrometry/mass spectrometry (LC–MS/MS). A major protein bound to the DARPP-32 matrix, but not to casein, and eluted by 150 mM NaCl was identified as a mixture of α- and β-adducin (Supplementary Fig. 1a,b). To confirm this finding, eluates from casein- or DARPP-32-Sepharose columns were immunoblotted for β-adducin (Fig. 1a). The DARPP-32:β-adducin interaction was further confirmed by the precipitation of β-adducin by anti-green fluorescent protein (GFP) antibody in striatal lysates from DARPP-32-GFP transgenic mice but not from wild-type (WT) littermates (Fig. 1b,c).

Adducins comprise three isoforms (α, β and γ, or adducin 1–3), which form α/β or α/γ heterotetramers that cap the fast-growing ends of actin filaments and link them to the spectrin cytoskeleton^19^. α- and γ-adducin are ubiquitous, whereas β-adducin is expressed predominantly in brain and erythrocytes^20^. α- and β-adducins are highly expressed in the brain, including the striatum^21^. Adducins comprise an N-terminal globular domain, a neck linker region and a conserved C-terminal MARCKS domain^19^ (named after the myristoylated alanine-rich C-kinase substrate in which it was first identified^22^; Fig. 1d). To pinpoint the domain of β-adducin responsible for the interaction with DARPP-32, myc-tagged β-adducin or its fragments (Fig. 1d) were immobilized and then loaded with lysates from COS-7 cells expressing DARPP-32-GFP. Bound proteins were eluted and immunoblotted with β-adducin and DARPP-32 antibodies (Fig. 1e). DARPP-32-GFP interacted with all fragments of β-adducin that contained the C-terminal MARCKS domain (residues 691–726) as well as with the full-length protein, but not with a 502–613-fragment lacking the MARCKS domain (Fig. 1e,f). These results indicated that β-adducin binds to DARPP-32 through its MARCKS domain.

### DARPP-32 phosphorylation modulates β-adducin binding

**Figure 2:**
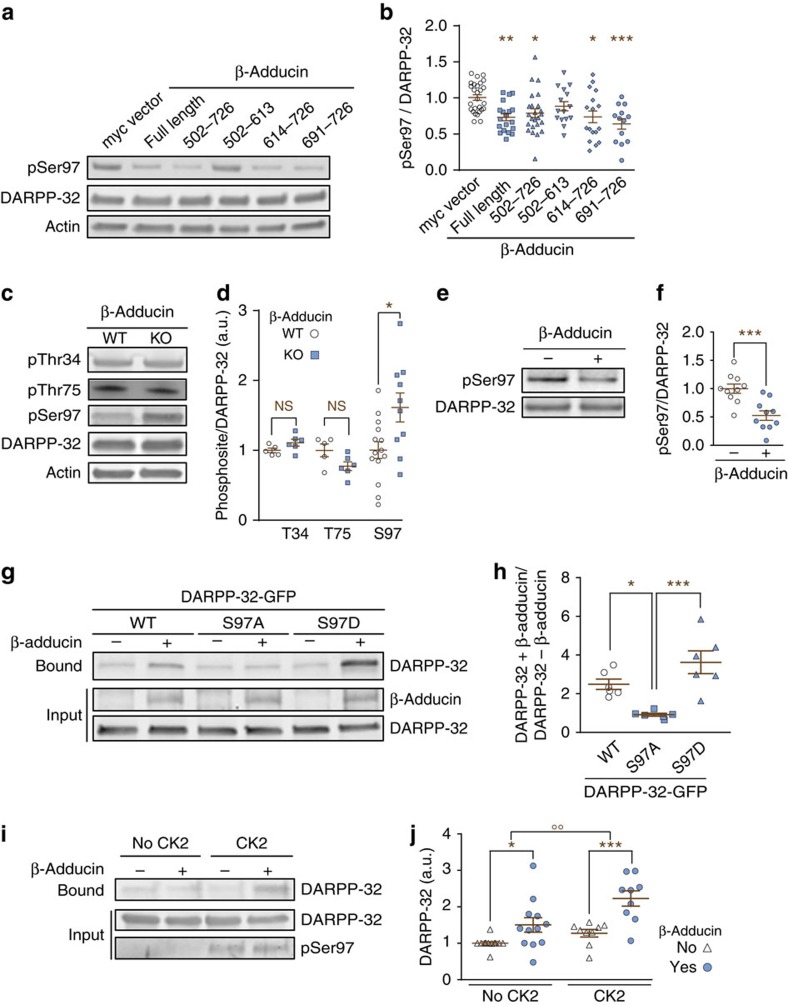
DARPP-32 Ser97 phosphorylation enhances β-adducin interaction. (**a**) COS-7 cells were co-transfected with DARPP-32-GFP and full-length myc-β-adducin or its fragments. PhosphoSer97-DARPP-32 (pSer97), total DARPP-32 and actin as loading control were analysed by immunoblotting. (**b**) Quantification of results as in **a**. *n*=13–28 per group from >3 experiments; one-way ANOVA: *F*_(5,114)_=5.1, *P*<0.001; Tukey’s test versus myc vector: *P<0.05; **P<0.01; ***P<0.001. (**c**) Phosphorylation of DARPP-32 Thr34, Thr75 and Ser97 (pThr34, pThr75 and pSer97, respectively), total DARPP-32 and actin were analysed by immunoblotting striatal lysates from WT or β-adducin KO mice. (**d**) Quantification of results as in **c**. *n*=5–14 mice per group from two experiments; Student’s *t*-test, pThr34, *t*_8_=1.68, *P*=0.13; pThr75, *t*_8_=1.70, *P*=0.13; pSer97, *t*_22_=2.71, *P=0.013; NS, not significant. (**e**) Purified DARPP-32 and CK2α_2_β_2_ were incubated with 100 μM ATP at 37 °C for 30 min in the absence (−) or presence (+) of purified β-adducin. pSer97 and total DARPP-32 were analysed by immunoblotting. (**f**) Quantification of results as in **e**. *n*=10 per group from >3 experiments; Student’s *t*-test, *t*_18_=4.04, ***P<0.001. (**g**) Beads coated or not with β-adducin were incubated with lysates of COS-7 cells transfected with WT, S97A or S97D-DARPP-32-GFP. DARPP-32 was detected in β-adducin precipitates by immunoblotting. (**h**) Quantification of results as in **g** as ratios of DARPP-32 precipitated in the presence (+)/absence (−) of β-adducin. *n*=6 per group; one-way ANOVA: *F*_(2,15)_=14.26; *P*<0.001. Tukey’s test versus S97A: *P<0.05; ***P<0.001. (**i**) Beads coated (+) or not (−) with β-adducin were incubated for 2 h at 4 °C with purified recombinant DARPP-32 phosphorylated or not by CK2, as indicated. After washing, DARPP-32 bound to the beads, and DARPP-32 and pSer97 in the input were analysed by immunoblotting. (**j**) Quantification of data as in **i**. *n*=9–12 per group from >3 experiments; two-way ANOVA: β-adducin factor, *F*_(1,38)_=22.18, *P*<10^−4^; CK2 phosphorylation factor, *F*_(1,38)_=10.41, *P*=0.003; no interaction, *F*_(1,38)_=2.14; Šidák’s test versus no β-adducin, *P<0.05, ***P<0.001; phospho versus non-phospho, °°*P*<0.01. In **b**,**d**,**f**,**h**,**j**, independent data points are plotted and means±s.e.m. are shown.

Since DARPP-32 is an intrinsically disordered protein with little identifiable secondary structure^23^,^24^, a useful read-out of its interactions in cells is the status of its phosphorylation sites. We noticed that in COS-7 cells co-transfected with DARPP-32 and myc-tagged β-adducin constructs, the presence of β-adducin reduced Ser97 phosphorylation of DARPP-32, whereas Thr34 and Thr75 phosphorylation were not altered (Supplementary Fig. 2a,b). The decrease in pSer97 was observed with all fragments of β-adducin that contained the MARCKS domain (Fig. 2a,b), in agreement with the binding data *in vitro*. To study whether Ser97 phosphorylation was also affected by the presence of β-adducin in the brain, we analysed β-adducin knockout (KO) mice^20^. In striatal lysates from mutant mice, Ser97 phosphorylation was increased as compared with WT, whereas phosphorylation of Thr34 and Thr75 was not significantly altered (Fig. 2c,d), showing that β-adducin has a negative effect on DARPP-32 phoshorylation on Ser97 *in vivo*.

This effect of β-adducin could occur through recruitment or stimulation of a phosphatase acting on pSer97, blockade of Ser97 access to its kinase CK2 (ref. 25) or by direct inhibition of CK2. We ruled out the possible role of a phosphatase because okadaic acid, a potent inhibitor of protein phosphatase-2A, the most active phosphatase for pSer97 (ref. 25), did not prevent the β-adducin-induced reduction in pSer97 (Supplementary Fig. 2c,d). To determine whether β-adducin inhibits Ser97 phosphorylation directly, we incubated purified DARPP-32 with CK2 and ATP in the presence or absence of β-adducin. Phosphorylation of Ser97 by CK2 was significantly diminished in the presence of β-adducin (Fig. 2e,f). In contrast, the β-adducin-binding partner calmodulin, used as a control, did not alter Ser97 phosphorylation by CK2 (Supplementary Fig. 2e,f). Moreover, β-adducin did not modify the phosphorylation of calmodulin by CK2 on Ser79/Thr81 (ref. 26), showing that β-adducin is not a general inhibitor of CK2 (Supplementary Fig. 2g,h). Taken together, these findings suggest that β-adducin reduces phosphorylation of DARPP-32 Ser97 by directly masking this site and thus that the region of interaction with β-adducin includes Ser97.

We then examined whether Ser97 phosphorylation had an impact on DARPP-32:β-adducin interaction. Lysates from cells transfected with myc-tagged β-adducin or an empty myc vector were immobilized to myc-affinity resin and incubated with WT DARPP-32 or mutants in which Ser97 was replaced by alanine or aspartic acid (Fig. 2g,h). β-Adducin co-precipitated with WT DARPP-32 and with S97D-DARPP-32, a phosphomimetic mutant, whereas it did not interact with non-phosphorylatable S97A-DARPP-32 (Fig. 2g,h). These results suggested that phosphorylation of Ser97 could enhance the interaction of DARPP-32 with β-adducin. *In vitro* assays supported these findings. Immobilized myc-β-adducin was incubated with purified recombinant DARPP-32 phosphorylated or not by CK2. Phosphorylation of DARPP-32 by CK2 further increased its binding to β-adducin (Fig. 2i,j). Hence, Ser97 phosphorylation enhances DARPP-32 binding to β-adducin although it is not required for the interaction. Altogether these experiments indicate that the interaction of β-adducin with unphosphorylated DARPP-32 is sufficient to impair access of Ser97 to CK2. We cannot exclude that, in specific conditions, stronger binding of β-adducin to pSer97-DARPP-32 may prevent its dephosphorylation. However, the inhibitory effects of β-adducin on DARPP-32 phosphorylation appear predominant in transfected cells in culture, as well as in striatal neurons *in vivo*, in which pSer97 was increased in β-adducin KO mice. Thus, our observations identify a key role for the region of DARPP-32 encompassing Ser97 in the interaction with the MARCKS domain of β-adducin.

### β-adducin interacts with DARPP-32 in striatal neurons

**Figure 3:**
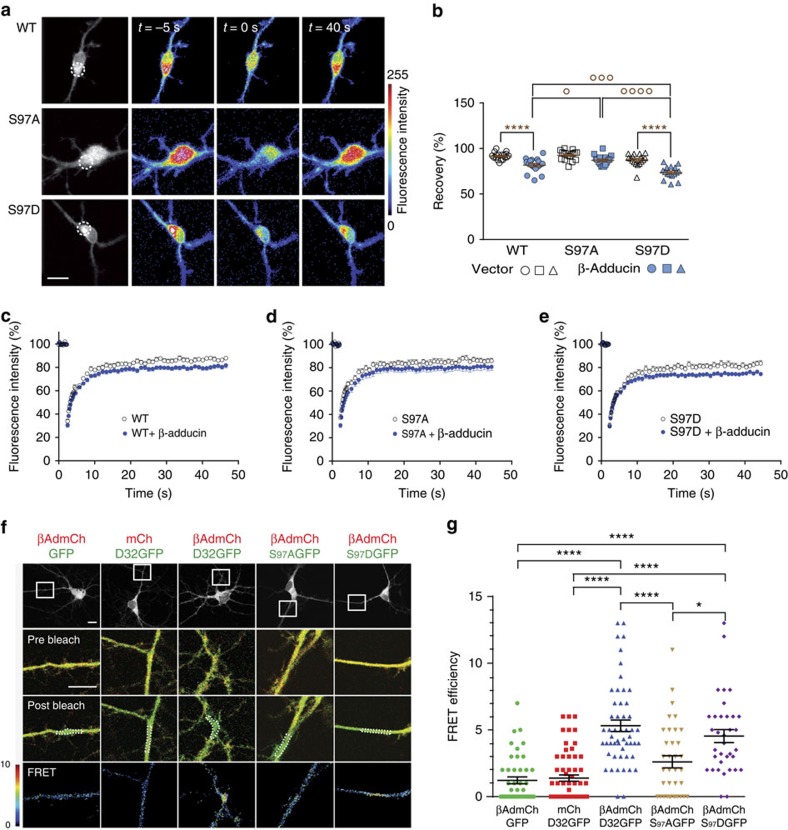
β-adducin alters DARPP-32 mobility and interacts with it in neurons. (**a**) Mouse striatal neurons in culture were transfected with WT, S97A or S97D-DARPP-32-GFP and fluorescence monitored before and after photobleaching (photobleached area, dotted line). Scale bar, 10 μm. (**b**–**e**) GFP fluorescence was recorded in cells co-transfected with myc vector (open bars or symbols) or myc-β-adducin (blue bars or symbols). (**b**) Quantification of average recovery (30–60 s). Independent data points are plotted and means±s.e.m. are shown, *n*=14–18 per group from 3 experiments; two-way ANOVA: β-adducin factor, *F*_(1,95)_=65.47, *P*<10^−4^; Ser97 mutation factor, *F*_(2,95)_=19.82, *P*<10^−4^; interaction, *F*_(2,95)_=4.01, *P*=0.02. Šidák’s test, presence versus absence of β-adducin, *P<0.05, ****P<10^−4^; effects of Ser97 mutation in the presence of β-adducin, °*P*<0.05; °°°°*P*<10^−4^. (**c**) Time course of recovery after photobleaching of WT DARPP-32-GFP (WT) fluorescence, in the absence or presence of co-transfected β-adducin. Data are means ± s.e.m. Two-way ANOVA: effect of β-adducin, *F*_(1,1947)_=198.6, *P*<10^−4^; time factor, *F*_(58,1947)_=177.2, *P*<10^−4^; no interaction, *F*_(58,1947)_=1.15. (**d**) Same as in **c** for S97A-DARPP-32-GFP. Two-way ANOVA: effect of β-adducin, *F*_(1,1740)_=91.56, *P*<10^−4^; time factor, *F*_(59,1740)_=76.94, *P*<10^−4^; no interaction, *F*_(58,1947)_=0.45. (**e**) Same as in **c** for S97D-DARPP-32-GFP. Two-way ANOVA: effect of β-adducin, *F*_(1,1800)_=135.1, *P*<10^−4^; time factor, *F*_(59,1800)_=135.0, *P*<10^−4^; no interaction, *F*_(58,1947)_=1.31. (**f**) For acceptor photobleaching, striatal neurons in culture were co-transfected as indicated with mCherry (mCh) or β-adducin–mCherry (βAdmCh), and GFP or GFP fused to either WT DARPP-32 (D32GFP) or S97A-DARPP-32 (S97AGFP) or S97D-DARPP-32 (S97DGFP). Top row, low-magnification single confocal sections indicating the area blown up in bottom rows (rectangles). Bottom rows, pre-bleach, post-bleach and calculated FRET images, respectively. Photobleached areas are indicated by dotted lines. FRET efficiency was calculated by measuring GFP fluorescence before and after mCherry photobleaching (pseudocolour scale indicated at bottom left). Scale bars, 10 μm. (**g**) Quantification of FRET efficiency as in **f**. Independent data points are plotted and means±s.e.m. are shown. Statistical analysis, one-way ANOVA: *F*_(4,223)_=26.97; *P*<0.0001. Tukey’s test: *P<0.05; ****P<10^−4^.

We next examined whether the DARPP-32:β-adducin interaction occurs in neurons. Since β-adducin is associated with the actin–spectrin cytoskeleton, whereas DARPP-32 is soluble, we examined whether the presence of β-adducin could influence the mobility of DARPP-32. We used fluorescence recovery after photobleaching (FRAP) in mouse striatal neurons in primary culture, transfected with WT, S97A- or S97D-DARPP-32-GFP (Fig. 3a). At 7 days in culture, the low levels of endogenous β-adducin allowed comparison of the mobility of DARPP-32 in the presence (transfection with β-adducin) or absence (transfection with empty vector) of β-adducin. In the absence of β-adducin, there was no difference in the fluorescence recovery rate of the DARPP-32-GFP mutants compared with WT DARPP-32-GFP (Fig. 3b–e). Expression of β-adducin decreased the fluorescence recovery of WT DARPP-32, indicating that β-adducin decreased the mobility of DARPP-32 (Fig. 3b,c). Interestingly, this effect was larger with the phosphomimetic S97D-DARPP-32-GFP but less pronounced with S97A-DARPP-32-GFP (Fig. 3b–e), consistent with the enhancement of DARPP-32:β-adducin interaction by Ser97 phosphorylation observed *in vitro*. These data provide strong indirect evidence for an interaction of DARPP-32 with β-adducin in live neurons and support the enhancement of DARPP-32 binding to β-adducin Ser97 phosphorylation.

To obtain direct evidence of DARPP-32:β-adducin interaction we used acceptor photobleaching^27^ in neurons co-transfected with DARPP-32 fused to GFP and β-adducin fused to mCherry (Fig. 3f). In these experiments, the existence of fluorescence (Föster) resonance energy transfer (FRET) between the GFP and mCherry was used as an index of the proximity of the two fusion proteins. The FRET efficiency was higher in neurons co-transfected with DARPP-32-GFP and β-adducin–mCherry than in those co-transfected with either unfused mCherry or GFP (Fig. 3f,g). The FRET increase was markedly diminished when WT DARPP-32 was replaced with the S97A mutant (Fig. 3g). In contrast, the S97D phosphomimetic mutant gave a significant FRET (Fig. 3g). Together these experiments provide strong evidence that DARPP-32 and β-adducin interact in neurons and that DARPP-32 Ser97 is important for this interaction, which is presumably modulated by phosphorylation.

**Figure 4:**
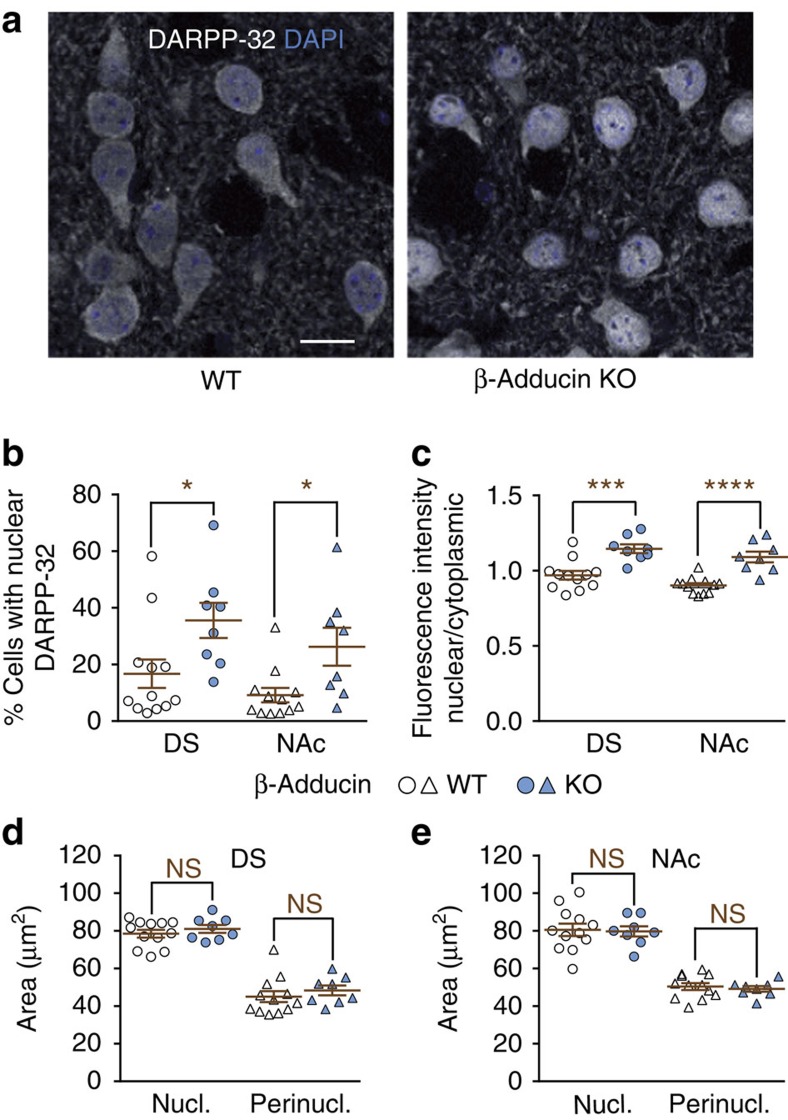
β-adducin influences DARPP-32 cytonuclear localization *in vivo*. (**a**) Dorsal striatum sections from WT or β-adducin KO mice were labelled with DARPP-32 antibody and nuclei were stained with DAPI. Scale bars, 10 μm. (**b**) The percentage of cells with DARPP-32 immunoreactivity intensity in the nucleus greater than or equal to the cytoplasm was quantified in the dorsal striatum (DS) or nucleus accumbens (NAc) of β-adducin KO mice and WT littermates in sections as in **a**. Independent data points are plotted and means±s.e.m. are shown, *n*=8–12 mice per group from 2 experiments; two-way ANOVA: region effect, *F*_(1,36)_=2.7, not significant (NS); genotype effect, *F*_(1,36)_=12.6, *P*<0.001; no interaction, *F*_(1,36)_=0.03; *post hoc* multiple comparison Šidák’s test, β-adducin KO versus WT, *P<0.05. (**c**) Same as in **b** except that the fluorescence intensity ratio between nucleus and cytoplasm was quantified. Two-way ANOVA: region effect, *F*_(1,36)_=4.98, *P*=0.03; genotype effect, *F*_(1,36)_=44.7, *P*<10^−4^; no interaction, *F*_(1,36)_=0.052; Šidák’s test, β-adducin KO versus WT, ***P<0.001, ****P<10^−4^. The nucleus (Nucl.) and perinuclear cytoplasm (Perinucl.) areas were measured in the DS (**d**) and NAc (**e**). No significant difference between genotypes was detected: two-way ANOVA: genotype effect, DS, *F*_(1,36)_=1.24, *P*=0.30; NAc, *F*_(1,36)_=0.16, *P*=0.69.

We have previously reported that DARPP-32 undergoes continuous cytonuclear trafficking regulated by phosphorylation of Ser97, which facilitates export from the nucleus^10^. Since adducins are predominantly in the cytoplasm, interaction with these proteins could contribute to DARPP-32 retention outside the nucleus. We investigated whether the absence of β-adducin altered DARPP-32 localization in striatal neurons *in vivo*. We measured DARPP-32 immunoreactivity in the nucleus and in the perinuclear cytoplasm, and compared the two values for each cell. In β-adducin KO mice the percentage of cells with predominantly nuclear DARPP-32 (that is, immunoreactivity in nucleus≥immunoreactivity in cytoplasm) was higher than in WT, in both the dorsal striatum and the nucleus accumbens (Fig. 4a,b). Accordingly, the nucleocytoplasmic fluorescence intensity ratio in the nucleus was increased (Fig. 4c). In contrast there was no change in the area of the nucleus and the perinuclear cytoplasm in the dorsal striatum (Fig. 4d) and in the nucleus accumbens (Fig. 4e). This result shows that β-adducin contributes to the retention of DARPP-32 in the cytoplasm *in vivo*, presumably through an interaction between the two proteins.

### DARPP-32 increases β-adducin phosphorylation on Ser713

**Figure 5:**
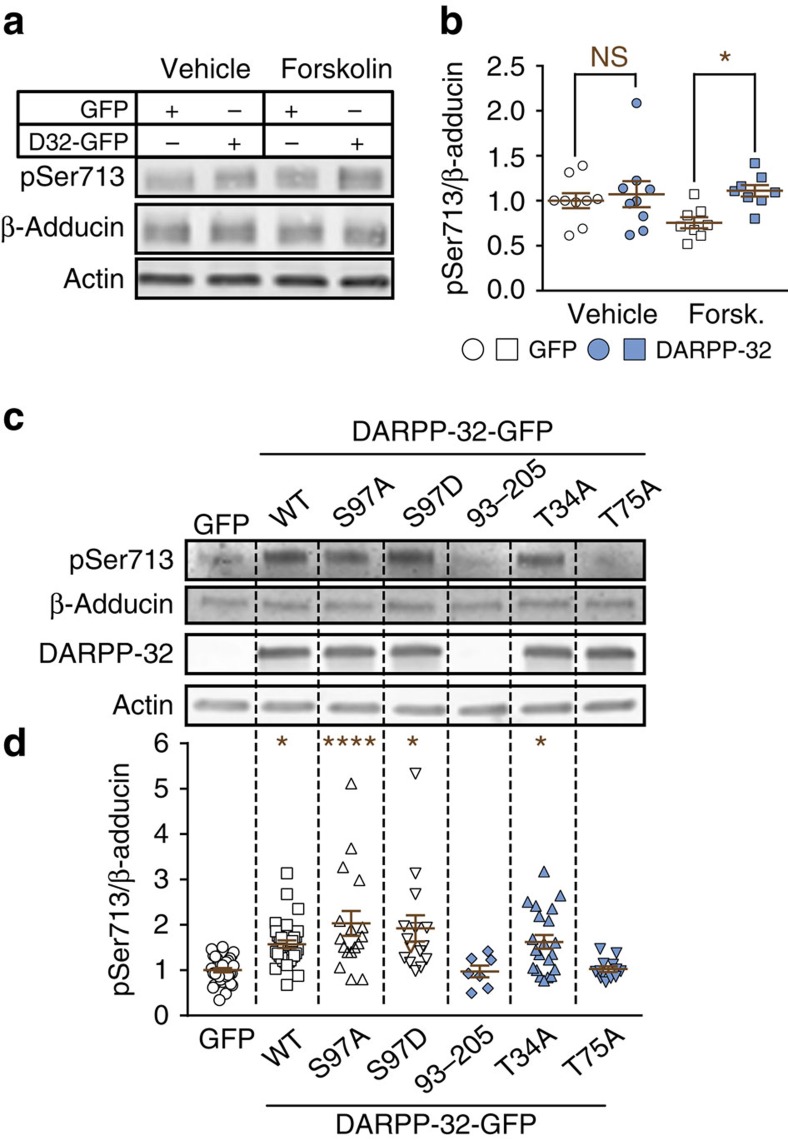
DARPP-32 increases β-adducin phosphorylation at Ser713. (**a**) COS-7 cells co-transfected with myc-β-adducin and either GFP or DARPP-32-GFP (D32-GFP) were incubated with forskolin (Forsk., 100 μM for 20 min) or vehicle (dimethyl sulfoxide 1/1,000 final). β-adducin pSer713, total β-adducin, DARPP-32 and actin as loading control were analysed by immunoblotting. (**b**) Quantification of results as in **a**. Independent data points are plotted and means±s.e.m. are shown; *n*=8–30 per group from >3 experiments; two-way ANOVA: DARPP-32 effect, *F*_(1,30)_=4.71, *P*=0.04; forskolin effect, *F*_(1,30)_=1.12, *P*=0.3; no interaction, *F*_(1,30)_=2.10; Šidák’s test, DARPP-32 versus GFP: *P<0.05. (**c**) COS-7 cells transfected with β-adducin and WT or various mutated forms of DARPP-32-GFP were treated with forskolin 100 μM for 20 min. β-adducin pSer713, total β-adducin, DARPP-32 and actin as loading control were analysed by immunoblotting. (**d**) Quantification of results as in **c**. Independent data points are plotted and means±s.e.m. are shown. *n*=7–40 per group from >3 experiments; one-way ANOVA: *F*_(6,132)_=7.68, *P*<10^−4^; Tukey’s test versus GFP: *P<0.05; ***P<0.001. NS, not significant.

DARPP-32 has a dual function in the regulation of signalling pathways by inhibiting protein phosphatase-1 when phosphorylated on Thr34 (ref. 8) and inhibiting PKA when phosphorylated on Thr75 (ref. 9). Since phosphorylation of Ser713 in the MARCKS domain of β-adducin by PKC prevents the formation of actin:spectrin complexes^28^ and regulates synapse stability^13^,^17^, we investigated whether DARPP-32 altered phosphorylation of Ser713. We expressed β-adducin in COS-7 cells in the presence of GFP or DARPP-32-GFP and monitored Ser713 phosphorylation. Stimulation of PKC with 12-*O*-tetradecanoylphorbol-13-acetate increased Ser713 phosphorylation as expected, with no effect of DARPP-32 (Supplementary Fig. 3a,b). We then tested the effects of stimulating the cAMP pathway in which DARPP-32 is mostly implicated^6^,^7^. In the absence of DARPP-32, incubation with forskolin, an activator of adenylyl cyclase, or with Sp5,6-DCl-cBIMPS (cBIMPS), a cAMP analogue, tended to reduce Ser713 phosphorylation (Fig. 5a,b and Supplementary Fig. 3c–f), presumably as a result of cAMP-dependent activation of protein phosphatase-2A (refs 10, 29; see further discussion below). In contrast, in the presence of DARPP-32 forskolin or cBIMPS increased Ser713 phosphorylation (Supplementary Fig. 3c–f).

We then investigated if any of the phosphorylation sites in DARPP-32 was involved in regulating the effect of forskolin on β-adducin Ser713 phosphorylation state. Mutations of DARPP-32 Ser97 did not alter the effects of forskolin on β-adducin (Fig. 5c,d). In contrast, truncation of the N-terminal region (residues 1–92) of the protein (DARPP-32_93–205_) resulted in a failure to observe the increase seen with intact DARPP-32 (Fig. 5c,d). We examined the role of the two phosphorylation sites situated in the N-terminal region of DARPP-32, Thr34 and Thr75. Surprisingly, mutation of Thr34, the site phosphorylated by PKA and responsible for protein phosphatase-1 inhibition^8^, did not prevent the effect of DARPP-32 on β-adducin phosphorylation, whereas T75A mutation abolished this effect (Fig. 5c,d). None of the DARPP-32 mutants altered β-adducin Ser713 phosphorylation in the absence of cAMP stimulation (Supplementary Fig. 3g).

Since PKA can stimulate protein phosphatase-2A (refs 10, 29), we hypothesized that this phosphatase could be involved in the regulation of β-adducin pSer713 by DARPP-32. In COS-7 cells co-expressing β-adducin and DARPP-32, okadaic acid, a preferential protein phosphatase-2A inhibitor^30^, abolished the forskolin-induced decrease in β-adducin phosphorylation on pSer713 and occluded the effect of DARPP-32 expression (Supplementary Fig. 3c,d). In contrast, tautomycetin, which is more active on phosphatase-1 than phosphatase-2A (ref. 31), did not occlude the effects of DARPP-32 (Supplementary Fig. 3h,i). The activity of tautomycetin was verified in this experiment by its ability to increase phosphorylation of histone H3 Ser10, a protein phosphatase-1 substrate (Supplementary Fig. 3j,k). Together these findings provide strong evidence that activation of the cAMP/PKA pathway can decrease β-adducin phosphorylation on Ser713 by activating protein phosphatase-2A, whereas phosphorylation of Thr75 in DARPP-32 likely opposes this effect by inhibiting PKA.

### DARPP-32 and caffeine-induced adducin phosphorylation

**Figure 6:**
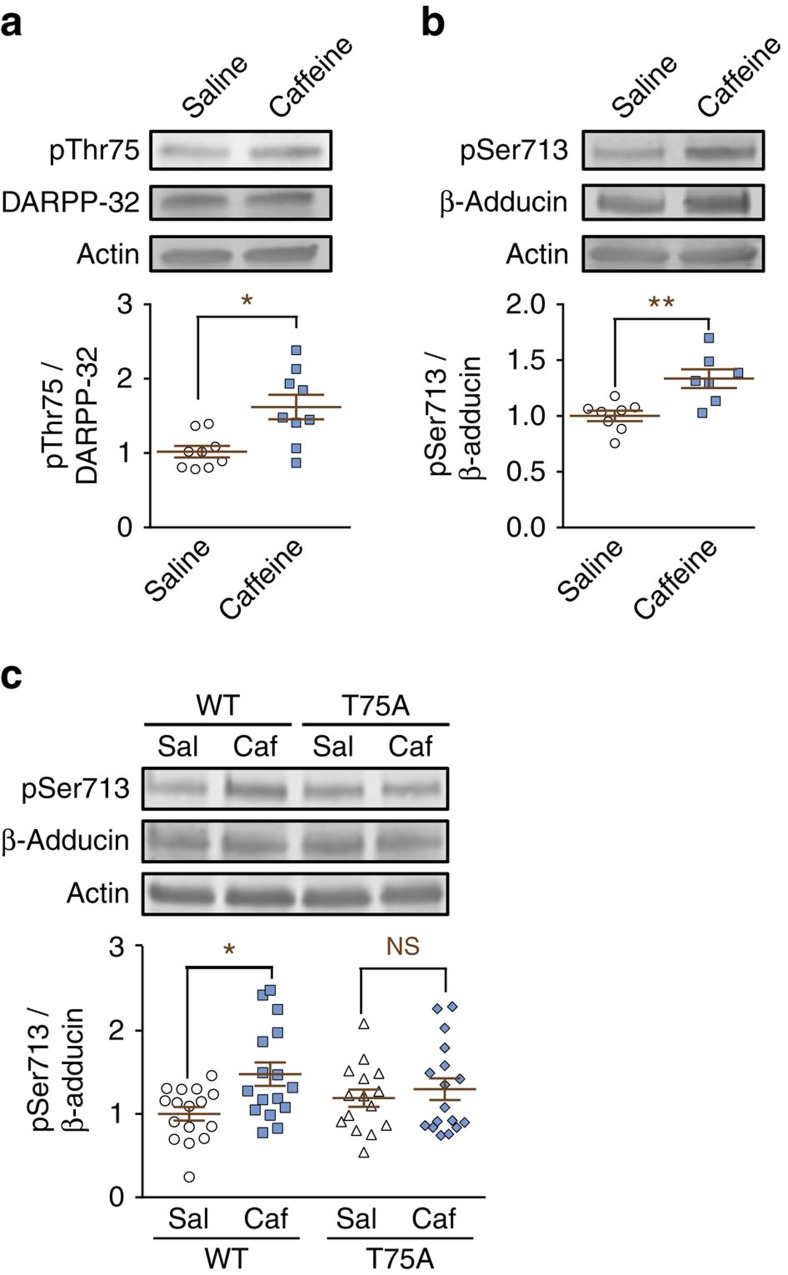
Caffeine stimulates adducin phosphorylation through DARPP-32. (**a**) Striatal samples were prepared from C57BL/6 mice 40 min after intraperitoneal injection of saline or 7.5 mg kg^−1^ caffeine. pThr75 and total DARPP-32, and actin as loading control, were analysed by immunoblotting. Lower panel, independent data points are plotted and means±s.e.m. are shown; *n*=9 per group from 2 experiments; Student’s *t*-test, *t*_16_=2.53, *P<0.05. (**b**) Samples obtained as in **a** were immunoblotted for pSer713 and total β-adducin and actin. Lower panel as in **a**, *n*=7 per group from 2 experiments; Student’s *t*-test, *t*_14_=3.66, **P<0.01. (**c**) T75A knock-in mutant mice or WT littermates were injected with saline or caffeine as in **a** and killed 40 min later. Striatal samples were analysed as in **b**. Lower panel as in **a**, *n*=12–15 per group from >3 experiments; two-way ANOVA: caffeine factor, *F*_(1,60)_=6.28, *P*=0.015; genotype factor, *F*_(1,60)_=0.001, not significant (NS); no interaction, *F*_(1,60)_=2.48. Šidák *post hoc* test, caffeine versus saline: *P=0.01.

To determine whether DARPP-32 regulates β-adducin phosphorylation through its Thr75 phosphorylation site *in vivo*, we tested the effects of caffeine administration, a mild psychostimulant previously shown to increase DARPP-32 Thr75 phosphorylation in mouse striatum^32^,^33^. We first confirmed that caffeine administration in C57BL/6 mice increased phosphorylation of DARPP-32 Thr75 under our experimental conditions (Fig. 6a). Caffeine also increased β-adducin Ser713 phosphorylation in the same samples (Fig. 6b). Importantly, the caffeine-induced increase in adducin phosphorylation was abolished in T75A mutant mice^34^ (Fig. 6c), indicating that phosphorylation of DARPP-32 at Thr75 is necessary for this effect *in vivo*. Thus, these results provide direct evidence for a role of the pThr75-DARPP-32/PKA/phosphatase-2A cascade in regulating adducin phosphorylation *in vivo*.

### β-adducin regulates actin filaments in striatal neurons

**Figure 7:**
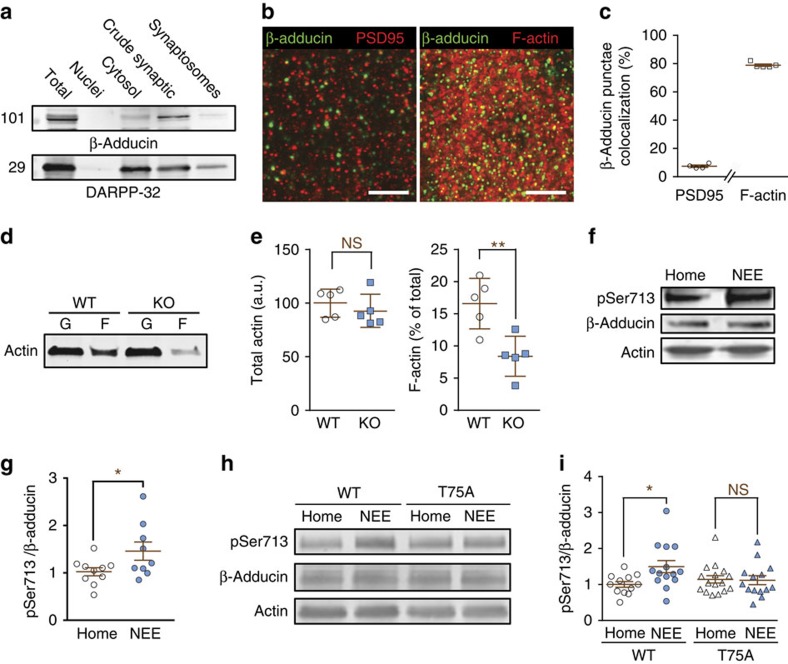
β-Adducin localization and phosphorylation in the striatum. (**a**) Striatal homogenate were separated into major subcellular fractions by differential centrifugation and β-adducin immunoreactivity detected by immunoblotting. (**b**) Striatal sections were labelled with antibodies for β-adducin (green) and PSD-95 (red, left panel) or phalloidin (F-actin, red, right panel). Scale bar, 5 μm. (**c**) Co-localization of β-adducin and PSD-95 or F-actin was quantified and expressed as the percentage of β-adducin aggregates, which overlapped with aggregates of the other marker. *n*=4–5. (**d**) Globular (G) and filamentous (F) actin were separated in striatal homogenates from WT or β-adducin KO mice. (**e**) Quantification of results as in **d**. Total actin (=F+G in **d**) was normalized to the mean of WT. Statistical analysis, unpaired Student’s *t-*test, total actin, *t*_8_=0.81, not significant (NS); F-actin, *t*_8_=3.67, **P<0.01. (**f**) Striatal homogenates were prepared from mice housed in their home cage or placed in a NEE for 24 h before sacrifice, and analysed by immunoblotting with antibodies for pSer713, β-adducin and actin as a loading control. (**g**) Quantification of results as in **f**, 9–10 mice per group from 2 experiments; Student’s *t*-test, *t*_17_=2.14, *P<0.05. (**h**) Striatal samples were prepared from WT or T75A mutant littermate mice housed in home cage or NEE, and analysed by immunoblotting as in **f**. (**i**) Quantification of results as in **h**, 11–15 mice per group from >3 experiments. Two-way ANOVA: genotype factor, *F*_(1,52)_=0.90, *P*=0.35; housing factor, *F*_(1,52)_=3.53, *P*=0.06; interaction, *F*_(1,52)_=4.30, *P*<0.05; Šidák *post hoc* test, NEE versus home cage, *P<0.05. Independent data points are plotted and means±s.e.m. are shown in **c**,**e**,**g**,**i**.

We then investigated the possible functional relevance of the pThr75-DARPP-32/β-adducin cascade. Adducin is enriched in dendritic spines^28^ and is involved in synapse formation and stabilization^13^,^17^,^35^, including in the formation of protrusions^17^ and spine stabilization^13^,^14^. We first examined the distribution of β-adducin in striatal tissue. Fractionation indicated that β-adducin was enriched in the crude synaptic fraction, but not in synaptosomes (Fig. 7a), which mostly comprise presynaptic elements and attached postsynaptic elements including postsynaptic densities (PSDs), suggesting its presence at the postsynaptic level but not particularly in PSDs. We then examined the co-localization of β-adducin immunoreactive punctae with PSD-95, a well-characterized PSD protein, and with filamentous actin (F-actin), which is highly enriched in striatal spines^36^ (Fig. 7b,c). We found that ∼10% of β-adducin immunoreactive punctae were co-labelled with PSD-95 and 80% with F-actin. These results indicated that in the striatum β-adducin is enriched in spines, in agreement with a previous report on α-adducin^21^, but that only a small fraction is closely associated with PSDs. We then examined whether β-adducin was important for F-actin regulation in the striatum by comparing the fraction of actin that was in the filamentous form in WT and β-adducin KO mice (Fig. 7d,e). F-actin was decreased by about twofold in the absence of β-adducin, whereas the total amount of actin was not altered, revealing the important role of β-adducin in the control of actin filaments dynamics in the striatum.

### NEE increases β-adducin phosphorylation

Since the interaction of β-adducin with actin cytoskeleton is regulated by phosphorylation, we investigated the possible regulation of β-adducin phosphorylation in the striatum in response to a physiologically relevant stimulus. In the hippocampus β-adducin phosphorylation is reported to increase following several weeks of environmental enrichment^13^, but the existence of earlier changes has not been examined. Since changes in dendritic spines have been reported in the cortex within days after training^37^,^38^, and in the striatum dozens of minutes after exposure to drug-associated cues^39^, we asked whether a short exposure to novel environment could alter β-adducin phosphorylation in the striatum. Exposure to NEE was done by housing mice for 24 h in a spacious enclosure, containing several novel objects. Social stress induced by isolation was avoided by transferring all littermates from one cage to the same NEE cage. β-adducin phosphorylation was increased after a 24-h NEE (Fig. 7f,g). The increase in phosphorylation observed in WT mice was blocked in their T75A mutant littermates (Fig. 7h,i). These results showed that a day in an enriched environment increases β-adducin phosphorylation and that this stimulation requires pThr75-DARPP-32.

### β-adducin and spine regulation by NEE

**Figure 8:**
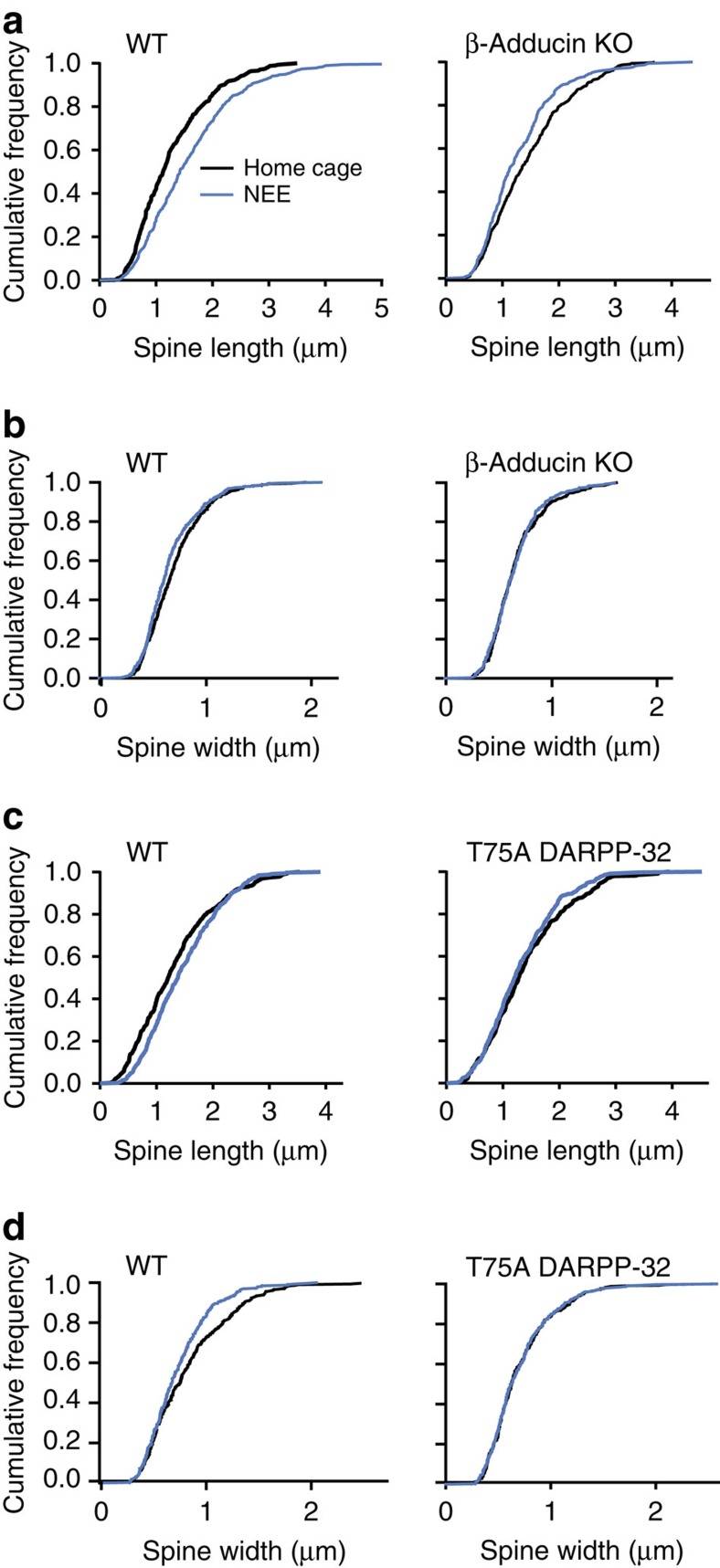
NEE alters dendritic spines in nucleus accumbens. WT and mutant mice were killed in their home cage or after 24 h in a NEE. Spines were analysed in the nucleus accumbens using the Golgi-Cox method (see Supplementary Fig. 6). (**a**) Spine length was measured in WT (left panel) and β-adducin KO (right panel) littermate mice (196–252 spines per group) and plotted as cumulative frequency. NEE increased spine length in WT but not in KO mice; Gehan–Breslow–Wilcoxon test: WT, *χ*^2^=32.92, degrees of freedom (DF)=1, *P*<10^−4^; KO, *χ*^2^=9.133, DF=1, *P*=0.0025. (**b**) Quantification of spine width in the same samples as in **a**, plotted as cumulative frequency. NEE increased spine width in WT but not in KO mice; Gehan–Breslow–Wilcoxon test: WT, *χ*^2^=5.992, DF=1, *P*=0.014; KO, *χ*^2^=0.04, DF=1, not significant (NS). (**c**) Analysis of spine length in WT (left panel) and Thr75Ala (T75A) DARPP-32 mutant littermate mice. NEE increased spine length in WT but not in T75A littermates; Gehan–Breslow–Wilcoxon test: WT, *χ*^2^=9.836, DF=1, *P*=0.0017; T75A, *χ*^2^=1.958, DF=1, NS. (**d**) Quantification of spine width in the same samples as in **c**, plotted as cumulative frequency. NEE increased spine width in WT but not in KO mice; Gehan–Breslow–Wilcoxon test: WT, *χ*^2^=13.78, DF=1, *P*=0.0002; KO, *χ*^2^=0.03, DF=1, NS; T75A, *χ*^2^=0.049, DF=1, NS.

We then investigated the effects of NEE on dendritic spines of nucleus accumbens medium-size spiny neurons using Golgi-Cox staining (Supplementary Fig. 4a). In home cage-housed WT mice our results were in good agreement with previous publications, with an average density of 1–1.5 spines per μm of dendrite^40^ and a distribution of thin>mushroom>stubby spines^41^. NEE increased the overall spine density in WT mice (Supplementary Fig. 4b). In β-adducin KO mice the total spine number was slightly decreased, and this was accounted for by a decrease in mushroom spines (Supplementary Fig. 4a–c), as previously reported^14^. The study of spine length in WT mice showed a clear effect of NEE, which shifted the distribution towards longer necks (Fig. 8a) with a small decrease in spine width (Fig. 8b). These effects disappeared in the absence of β-adducin, with even a trend to spine shortening in response to NEE (Fig. 8a,b). When spines were classified as stubby, thin and mushroom, NEE increased thin spines and decreased stubby spines, whereas these effects were absent in β-adducin KO mice (Supplementary Fig. 4c).

Since our results *in vitro* and *in vivo* showed that DARPP-32 is important for the regulation of β-adducin phosphorylation in striatal neurons (see above) and since this phosphorylation plays a key role in regulating β-adducin function^19^,^28^, we examined the consequences of DARPP-32 T75A mutation on spines’ responses to NEE. In the T75A mutant mice we found a slight decrease in total spine density, at the expense of stubby and mushroom spines, as compared with their WT littermates (Supplementary Fig. 5a–c). The overall increase in spine length and the small decrease in spine width induced by NEE in WT mice were absent in their DARPP-32 T75A littermates (Fig. 8c,d). The lack of effect of NEE in the T75A mutant mice was also apparent after spine classification (Supplementary Fig. 5c). Thus, the consequences of the absence of β-adducin and those of the DARPP-32 T75A mutation were similar, both preventing the effects of NEE on spine neck length. Since DARPP-32 T75A mutation also prevented the effects of NEE on β-adducin phosphorylation, this series of experiments strongly supports the role of the DARPP-32/β-adducin cascade in striatal spine plasticity.

### Effects of a NEE on cocaine response

**Figure 9:**
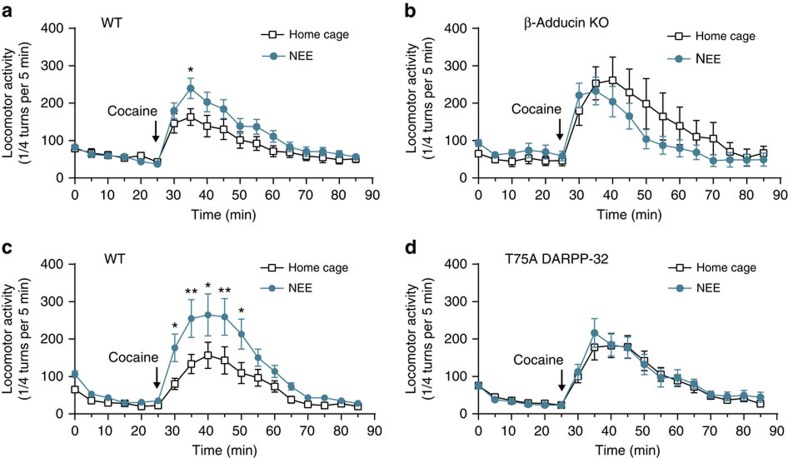
Role of β-adducin and DARPP-32 in NEE effects on cocaine response. WT (**a**) and β-adducin KO littermates (**b**) were housed for 24 h in their home cage or under NEE conditions, as indicated. After a 30-min habituation in the circular maze, all mice were injected with cocaine (arrow, 20 mg kg^−1^, intraperitoneal). Locomotor activity was recorded in 5-min bins. WT (**a**): 15 (home cage) and 17 (NEE) mice per group in 3 experiments; two-way ANOVA: housing factor, *F*_(1,540)_=16.54, *P*<10^−4^; time factor, *F*_(17,540)_=18.43, *P*<10^−4^; no interaction, *F*_(17,540)_=0.18, not significant (NS). β-adducin KO (**b**): 8 (home cage) and 11 (NEE) mice per group in 3 experiments; two-way ANOVA: housing effect *F*_(1,306)_=3.76, *P*=0.053; time factor, *F*_(17,306)_=9.53, *P*<10^−4^; no interaction, *F*_(17,306)_=0.55. (**c**,**d**) WT (**c**) and T75A littermates (**d**) were treated as in **a**. WT (**c**): 10 (home cage) and 11 (NEE) mice per group in 3 experiments; two-way ANOVA: housing factor, *F*_(1,324)_=38.56, *P*<10^−4^; time factor, *F*_(17,324)_=18.38, *P*<10^−4^; interaction, *F*_(17,324)_=1.85, *P*=0.02. T75A (**d**): 13 (home cage) and 13 (NEE) mice per group in 3 experiments; two-way ANOVA: housing factor, *F*_(1,432)_=0.40, NS; time factor, *F*_(17,432)_=20.47, *P*<10^−4^; no interaction, *F*_(17,432)_=0.20. (**a**–**d**) All data are means±s.e.m. (**a**,**c**) Šidák *post hoc* test for each time bin: *P<0.05; **P<0.01.

Prolonged enriched environment modifies responses to psychostimulant drugs^42^,^43^, including an increase in locomotor effects of a single injection^44^. However, the molecular mechanisms leading to these chronic effects of housing conditions are not known. Furthermore, it is not known whether brief exposure to NEE is sufficient to alter any response to psychostimulants. Since NEE altered spines, as described above, we first tested the existence of behavioural changes in the same time frame, that is, 24 h after exposure to enriched environment, using locomotor response to cocaine as global read-out. Locomotor activity induced by a single dose of cocaine was indeed increased in C57Bl/6 mice that had been housed in NEE, as compared with home cage-housed controls (Supplementary Fig. 6a,b). Since the response and differences were much more apparent at 20 mg kg^−1^, we used this dose in further experiments. We then tested the effects of NEE on cocaine response in β-adducin KO mice. We noted that, as previously reported^14^, cocaine-induced locomotion was enhanced in β-adducin KO mice compared with WT littermates (Supplementary Fig. 7a). However, whereas NEE increased cocaine-induced locomotion in the WT littermates, in β-adducin mutant mice NEE did not increase the response, and even tended to decrease it (Fig. 9a,b). These results showed that in the absence of β-adducin the effects of NEE were altered and suggested that β-adducin plays a role in the effects of NEE on cocaine-induced locomotion. We next investigated the possible role of the DARPP-32/β-adducin cascade in these effects of NEE using T75A-DARPP-32 mutant mice. The mutant mice had a slightly stronger locomotor response to cocaine than their WT littermates (Supplementary Fig. 7d). However, whereas NEE enhanced the cocaine-induced locomotor response in this group of WT mice (Fig. 9c), it had no effect in the T75A mutant littermates (Fig. 9d). Thus, our data show that a 24-h exposure to NEE increases the locomotion induced by a single cocaine injection, demonstrating the existence of functional alterations in the target circuits. Importantly, both pThr75-DARPP-32 and β-adducin are required for the ability of NEE to increase cocaine-induced locomotion, supporting the hypothesis that regulation of β-adducin phosphorylation through pThr75-DARPP-32 is implicated in this effect.

## Discussion

Our study identifies a direct interaction between DARPP-32 and β-adducin that has consequences for the properties of these proteins in striatal neurons. Our results in mice reveal similar morphological and behavioural consequences of β-adducin KO and of a point mutation in DARPP-32 that prevents β-adducin regulation, suggesting a functional role for the interaction between the two proteins *in vivo*. Moreover, our results show the existence of hitherto unknown, rapid effects of NEE on dendritic spines and drug responses, and provide evidence for a role of DARPP-32 and β-adducin in these effects.

At the molecular level, the interaction between DARPP-32 and β-adducin involves its positively charged MARCKS domain (13 basic residues among 26 C-terminal residues) and a negatively charged region of DARPP-32 (7 acidic residues among the 12 located just C-terminal to Ser97). The interaction of DARPP-32 with β-adducin was further enhanced by increasing its negative charge through phosphorylation of Ser97 by CK2 or its replacement by an aspartate. These results suggest that electrostatic bonds between negatively charged residues in DARPP-32 and positively charged residues in β-adducin MARCKS domain are involved in the interaction. Our results provide evidence that the interaction between the two proteins exists in striatal neurons since the presence of β-adducin decreases DARPP-32 mobility. Interaction between the two proteins in neurons was further supported by acceptor photobleaching FRET experiments, which confirmed the importance of Ser97 for the interaction. In addition, the presence of β-adducin alters the intracellular location of DARPP-32 *in vivo*. Thus, in addition to the previously reported role of Ser97 phosphorylation by CK2 to facilitate DARPP-32 nuclear export^10^, β-adducin appears to contribute to DARPP-32 intracellular localization, presumably by retaining it in the cytoplasm through direct binding.

**Figure 10:**
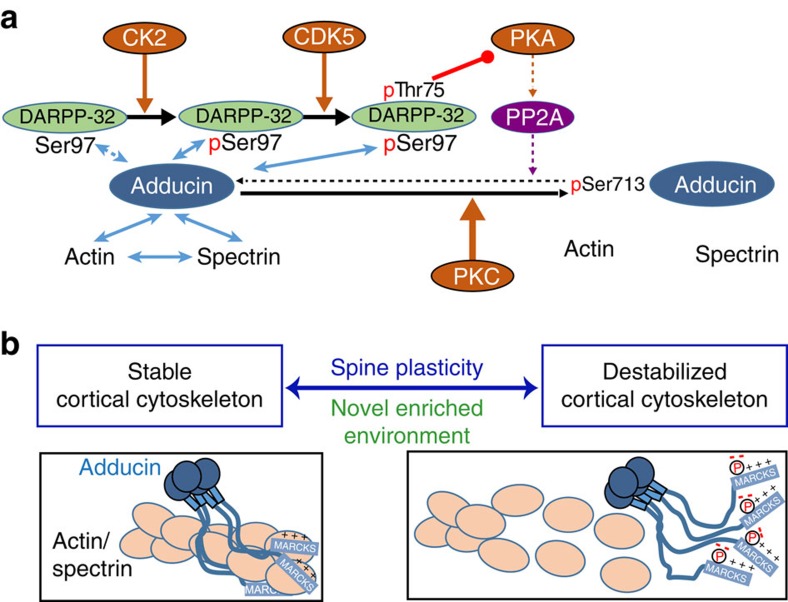
Model of DARPP-32:β-adducin interactions. (**a**) DARPP-32 can interact with β-adducin MARCKS domain and this interaction is increased by phosphorylation of DARPP-32 on Ser97 by CK2. When DARPP-32 is phosphorylated by CDK5 on Thr75 it inhibits PKA (red line with round end). Since PKA activates PP2A, which dephosphorylates β-adducin Ser713, pThr75-DARPP-32 increases β-adducin phosphorylation level (resulting from PKC action). (**b**) Phosphorylation of β-adducin prevents its interaction with actin and spectrin and destabilizes cortical cytoskeleton in dendrites and spines. This destabilization facilitates spine plasticity in response to NEE. Schematic representation of adducin tetramer and actin/spectrin is modified from ref. 19.

Although we used β-adducin to characterize the interaction with DARPP-32, α-adducin was also identified in striatal extracts in the initial DARPP-32 pull down. Since the MARCKS domain is highly conserved in sequence and function between adducin isoforms^19^, it is likely that DARPP-32 can interact with all these isoforms, which form heterotetramers in cells. As the MARCKS domain of adducins is necessary for their actin-binding, actin-capping and spectrin-recruiting activities^19^, DARPP-32 binding may directly influence these functions. However, our results identified an important effect of DARPP-32 through increased phosphorylation of β-adducin MARCKS domain on Ser713, a site also conserved in α- and γ-adducin. Our data in cells in culture and in mice indicate that pThr75-DARPP-32 prevents dephosphorylation of pSer713 (the involved pathways are summarized in Fig. 10). This can be accounted for by the inhibitory effect of pThr75-DARPP-32 on PKA^9^ and the resulting inhibition of a PKA-activated form of protein phosphatase-2A (refs 10, 29). Since phosphorylation of Ser713 by PKC disrupts adducin interactions with actin and spectrin^28^, our results predict that DARPP-32 plays a role in destabilizing these interactions (Fig. 10). Thus, modulation of adducin dephosphorylation represents a novel site for crosstalk between PKC and PKA pathways in striatal neurons, involving phosphorylation of DARPP-32 Thr75. This regulation is likely to exist *in vivo*, since the mild psychostimulant caffeine, which is known to increase DARPP-32 Thr75 phosphorylation^33^, also enhanced adducin phosphorylation (our study) and this effect was absent in DARPP-32T75A mutant mice.

DARPP-32 Thr75 is a substrate of Cdk5 (ref. 9), a protein kinase involved in dendritic spine and synapse formation through multiple mechanisms^45^,^46^,^47^ and necessary for the increase in spine density induced by chronic cocaine in medium-size spiny neurons^48^. Our results reveal a specific requirement of DARPP-32 Thr75 phosphorylation for the rapid effects of NEE on adducin phosphorylation. Since this phosphorylation disrupts adducin's interactions with actin and spectrin^28^, which are implicated in synapse formation and stabilization^13^,^17^, it may contribute to dendritic spines alteration rapidly induced by NEE. This hypothesis is supported by the blockade of specific NEE-induced spine alterations by either Thr75 mutation or β-adducin KO. Newly formed thin spines could contribute to increased plasticity^49^. Spine necks regulate biochemical and electrical signals through compartmentalization of Ca^2+^ and synaptic proteins, which could modulate synapse strength^50^,^51^. Increase in neck length might favour dopaminergic synapses, which in contrast to glutamatergic synapses, are situated predominantly at spine necks^2^. Chronic exposure to cocaine or methylphenidate increases spine length in nucleus accumbens neurons^40^. Spine alterations could be associated with altered behavioural sensitization^52^. In support of this hypothesis we found that DARPP-32 Thr75 and β-adducin are both necessary for the enhancement of locomotor response to cocaine following 24-h NEE. The combination of *in vitro* functional interaction between DARPP-32 and β-adducin and their similar contribution to the effects of NEE suggests a specific role for this interaction *in vivo*. Thus, our work reveals the existence of rapid effects of environmental enrichment on nucleus accumbens neurons and provides evidence for their molecular bases and functional consequences. Enriched environment is usually tested for long periods of time (several weeks or months)^5^, although a 24-h environmental enrichment is sufficient for improving some behavioural responses (for example, novel object recognition), whereas longer periods are necessary for other responses^53^. Changes in nucleus accumbens spines have been observed within the minute-to-hour range after exposure to drug-associated cues^39^,^54^. The early molecular and cellular changes that might underlie or precede the effects of enriched environment have received very little attention, although changes in histone post-translational modifications have been reported after as little as 3 h after environmental enrichment^55^. Thus, our results provide novel evidence about the pathways that can be operating at early times after exposure to enriched environment. Further work is needed to assess whether the responses we report are persistent. It will also be important to determine whether DARPP-32 and adducins are necessary for previously reported behavioural effects of long-term enriched environment in animal models of addiction and whether these changes are transferable to environmental effects in human drug abuse. Conversely, it is noteworthy that thin spines in nucleus accumbens neurons and cocaine behavioural effects are decreased in mouse models of depression^56^ and our preliminary evidence suggests that 24-h NEE mimics some antidepressant effects. Hence, the effects of NEE on depression phenotypes and the role of the DARPP-32/adducin regulation in the context of mood disorders will deserve further investigation.

In summary, this work shows the existence of rapid effects of environmental enrichment on dendritic spines of nucleus accumbens neurons and on the amplitude of the locomotor response to cocaine. We describe a novel molecular pathway involving DARPP-32 and adducin that is likely to contribute to these effects. Further work is needed to assess the role of this pathway in modifications induced in reward systems by long-term enriched environment.

## Methods

### Animals

All animal experiments were carried out on mice with the exception of the starting material for analysis on DARPP-32 affinity column, which was male rat striatum. Animals were housed in accordance with the ethical guidelines (Declaration of Helsinki and NIH, publication no. 85-23, revised 1985; the European Community Guidelines; and the French Agriculture and Forestry Ministry guidelines for handling animals, decree 87849, license A 75-05-22) and approved by the *Institut du Fer à Moulin* ethical committee. Male C57Bl/6 mice and Sprague–Dawley rats were purchased from Janvier and used at 10–12 weeks. The following mutant mice were studied: DARPP-32 knock-in mutants—T34A, T75A and S97A mutants^34^; DARPP-32-GFP BAC transgenic mice; and β-adducin KO mice^20^, and their respective WT littermates. For mutant mice male and female mice were used at 9–18 weeks, in balanced numbers between groups. No effect of sex was detected. For C57Bl/6 mice only males were studied. DARPP-32-GFP mutants were generated by the *Institut Clinique de la Souris*, Illkirch France by injecting a bacterial artificial chromosome (clone gensat RP23-132E9) including mouse DARPP-32 gene sequence in which the GFP sequence had been inserted by homologous recombination at the C-terminus of DARPP-32, into oocytes of a C57Bl/6J × SJL hybrid background. They were backcrossed for >10 generations in a C57Bl/6 N background. Commercially supplied C57Bl/6 mice and mutant mice were 2–3 and 2–4 months of age during experiments, respectively. Mice were housed in groups of 2–5. For NEE, the whole cage group was transferred into the NEE cage.

### DARPP-32 affinity column and mass spectrometry

A unit of 500 μg purified DARPP-32 (ref. 57) or casein (Sigma) was coupled to 200 μl of settled N-hydroxysuccinimide-activated Sepharose (GE Healthcare) according to the manufacturer’s instructions. Two rat striata were dissected and homogenized in 2 ml lysis buffer (50 mM Tris-HCl, pH 7.5, 0.5% Triton X-100 v/v, 1 mM dithiothreitol, 2 mM EDTA, protease inhibitor cocktail complete (Roche)), then sonicated three times for 30 s. To remove debris, the lysate was spun for 10 min at 13,000 r.p.m. and the pellet discarded. The lysate was split into two and precleared for 1 h at 4 °C on a rotor with untreated Sepharose. Next, one-half of the lysate was incubated for 2 h at 4 °C with the casein-bound Sepharose slurry, and the other half was incubated with the DARPP-32-bound slurry. Unbound lysate was removed and the resin was washed 10 times with 1 ml lysis buffer. Between each step, the slurry was collected by centrifugation at 1,000 r.p.m. for 3 min. Bound proteins were eluted for 30 min with a salt step gradient of 500 μl lysis buffer including 50, 100, 150 and 300 mM NaCl. Fractions were then lyophilized, resuspended in 2 × Laemmli buffer (see below), electrophoresed on a 4–12% Nu-page gel (Invitrogen) and stained with Bio-Safe Coomassie G-250 (Bio-Rad). Bands that occurred in the eluates from the DARPP-32-coupled but not in the casein-coupled column were excised and subjected to mass spectrometry analysis. Proteins isolated in one-dimensional SDS–polyacrylamide gels were subjected to *in situ* enzymatic digestion following standard protocols. Briefly, the gel bands were washed with 250 μl 50% acetonitrile/50% water for 5 min followed by 250 μl of 50 mM ammonium bicarbonate/50% acetonitrile/50% water for 30 min and a final wash of 10 mM ammonium bicarbonate/50% acetonitrile/50% water for 30 min. The gel bands were then dried in a Speedvac and rehydrated with 40 μl 0.0067 μg μl^−1^ trypsin (Promega Seq. Grade Mod. Trypsin, #V511X) and incubated at 37 °C for 16 h. LC–MS/MS was performed on a Thermo Scientific LTQ Orbitrap XL equipped with a Waters nanoACQUITY UPLC system. Peptides were loaded on a Waters Symmetry C18 180 μm × 20 mm trap column at 15 μl min^−1^, 99% buffer A (100% water with 0.1% formic acid) for 1 min. Peptide separation was performed on a Waters 1.7 μm, BEH130 C18, 75 μm × 250 mm nanoACQUITY UPLC column (35 °C) at 300 nl min^−1^ with buffer A (water with 0.1% formic acid) and buffer B (CH_3_CN with 0.1% formic acid). A linear gradient (51 min) was run with 5% buffer B at initial conditions, 50% B at 50 min, and 85% B at 51 min. Database searching LC–MS/MS data was searched using Mascot Distiller and the Mascot search algorithm (Matrix Science). Search parameters were variable methionine oxidation and propionamide (cysteine) with a peptide tolerance of +20 p.p.m., and MS/MS fragment tolerance of +0.6 Da, peptide charges of +2 or +3, and normal and decoy database searches; max. number of miscleavages: 1; variable modifications: oxidation (M), propionamide (C). Cutoffs were set to a protein coverage of at least 10%; expectation: <0; Rattus norvegicus. Obvious contaminants such as keratin were excluded from analysis.

### Antibodies, drugs and chemicals

Primary antibodies were: mouse monoclonal for DARPP-32 5a and 6 (ref. 58; 1/5,000), calmodulin (Upstate Cell Signaling # 05–173); rabbit polyclonal for pThr34, pThr75 and pSer97 (#12438, #2301, #3401; 1:2,000; Cell Signaling); total β-adducin (#AP20545PU-N, 1:500; Santa Cruz, Acris); pSer713 of β-adducin (also reacts with pSer662 of γ-adducin and pSer724 of α-adducin; #05-587; 1:1,000; Chemicon/Millipore); β-actin (#A5316; 1:1,000; Sigma); myc-tag (#05-724; 1:500; Millipore); GFP (Abcam #ab6556, 1/1,000); pThr79-pSer81-calmodulin (Abcam, #ab194526); and pSer10 histone H3 (#06-570; rabbit; Millipore). Secondary antibodies comprised of IRDye800-conjugated anti-mouse and anti-rabbit (#610-132-121, #611-132-122; both 1:4,000; Rockland) antibodies for immunoblotting and anti-mouse CY3-conjugated antibody (#A10521; 1:400; Molecular Probes) for immunochemistry. COS-7 cells were treated with one or the combination of the following compounds, as indicated, or with dimethyl sulfoxide vehicle: Sp 5,6-DCl-cBIMPS (10 μM; BIOLOG Life Science); forskolin (100 μM; Sigma); tetradecanoylphorbol-acetate (100 nM; Sigma); okadaic acid (200 nM; Sigma); and tautomycetin (10 nM; Tocris). Mice were injected intraperitoneally with the following drugs or with their vehicle (9 g l^−1^ NaCl) caffeine (7.5 mg kg^−1^, Sigma) and cocaine (10 or 20 mg kg^−1^, Coper).

### Cell culture and transfection

COS-7 cells (American Type Culture Collection) were cultured in six-well plates (10^6^ per well) in Dulbecco’s mimimal essential medium (DMEM, Invitrogen) with 10% fetal bovine serum. They were co-transfected using Lipofectamine 2000 in OptiMEM (both Invitrogen) and DARPP-32 and β-adducin vectors, both 5 μg DNA, or empty vectors. All DARPP-32 vectors contained a GFP reporter and a kanamycin-resistance gene (Clontech). All GFP proteins/constructs used in this study were eGFP. Full-length DARPP-32, T34A, T75A, S97A and S97D point mutations as well as the DARPP-32 fragment DARPP-32_93–205_ were utilized^10^. The latter was generated from WT-DARPP-32-EGFP by restriction of DARPP-32 with Xho1 and Pst1 (New England Biolabs) and insertion of the C-terminal 93–205 fragment into a N1-EGFP vector (Clontech). Vectors expressing the human form of full-length β-adducin carried a myc-tag and an ampicillin-resistance gene (pCMV-myc vector, Clontech). The C-terminal fragment of β-adducin (aa 502–726) was subcloned into pCMV-myc and served to generate the shorter fragments by PCR using EcoRI and Kpn restriction sites for insertion. The empty GFP and myc vectors were used as controls during co-transfection. After 4 h of transfection, the medium was replaced with serum-free DMEM. Cells were treated 24 h later in serum-free DMEM and collected in RIPA buffer (20 mM Tris-HCl, pH 7.4, 150 mM NaCl, 1 mM EDTA, 1% NP-40 (v/v), 10% glycerol (v/v), 1 g l^−1^ SDS, 0.5 g l^−1^ deoxycholate, 1 mM sodium orthovanadate and 50 mM NaF) and complete protease inhibitors (Roche), and prepared for immunoblot analysis by heating the samples in Laemmli buffer (100 mM Tris-HCl, pH 6.8, 12% glycerol, 40 g l^−1^ SDS, 2% (v/v) β-mercaptoethanol and bromphenol blue) for 10 min at 98 °C.

### Co-immunoprecipitation

For sequential co-immunoprecipitation, β-adducin and DARPP-32 were separately expressed in COS-7 cells. First, lysates from cells transfected with the myc constructs (full-length β-adducin, fragments of it or the empty myc vector) were lysed in RIPA buffer (see above), precleared by centrifugation at 13,000 r.p.m. for 10 min and incubated with anti-myc-coupled agarose (Pierce) for 4 h at 4 °C under agitation. Negative controls consisted of resin incubated with non-transfected lysate. Afterwards, the lysate was removed by centrifugation and the resin was incubated with precleared lysate from COS-7 cells transfected with DARPP-32. Lysate from the same batch of DARPP-32-transfected cells were equally distributed on the control- and myc-construct-containing columns and incubated O/N at 4 °C. The next day, the resin was washed 10 times in Tris-buffered saline with 1% Tween 20 containing an additional 50 mM NaCl, eluted in buffer provided by the manufacturer (Pierce) and subjected to immunoblot analysis against total DARPP-32.

Co-immunoprecipitation with DARPP-32-GFP mutants was performed using a GFP-trap kit (Chromotek) according to the supplier’s instructions. Briefly, striata from DARPP-32-GFP mutant mice and WT littermates were dissected on ice and homogenized in RIPA buffer (see above). Lysates were precleared by centrifugation at 13,000 r.p.m. for 10 min. The supernatants were incubated with the resin for 2 h at 4 °C under agitation, then rinsed 4 × in 250 μl washing buffer provided by the supplier and eluted with Laemmli buffer before being processed for immunoblotting for total β-adducin.

### Protein purification and *in vitro* phosphorylation assays

Purified β-adducin (human sequence) was generated in *Escherichia coli* with a 6xHis construct (Topo PCR T7/NT construct, Invitrogen). Expression from an O/N culture (optical density (OD)=0.4) was induced by 1 mM isopropyl-β-D-thiogalactoside for 6 h, after which bacterial pellets were collected by centrifugation for 30 min at 2,000 r.p.m. Pellets from 50-ml culture were lysed in 1 ml lysis buffer (50 mM sodium dihydrogen phosphate, 300 mM NaCl, 10 mM imidazole and 1 mg ml^−1^ lysozyme; pH 8.0), sonicated for 2 × 10 s and incubated for 30 min on ice. Next, lysates were cleared by centrifugation for 10 min at 13,000 r.p.m. and the His-tagged protein was enriched using pre-equilibrated Ni-NTA spin columns (Qiagen) according to the manufacturer's instructions. Eluted fractions were desalted and concentrated on amicon ultra-2 centrifugal filter devices 50K (Millipore).

Purified β-adducin (final concentration: 1 μM), DARPP-32 (1 μM), CK2α_2_β_2_ (100 ng per assay; New England Biolabs) and calmodulin (1 μM Sigma) were used for *in vitro* assay of Ser97 phosphorylation of DARPP-32. DARPP-32 and β-adducin, negative control or calmodulin were preincubated for 5 min in CK2 buffer (final concentration 50 mM HEPES, 20 mM MgCl_2_, 150 mM NaCl and 100 μM ATP; pH 7.5). Then, CK2α_2_β_2_ was added and the samples were incubated for 30 min at 30 °C and subsequently immunoblotted for pSer97 and total DARPP-32.

### Striatal neuronal culture

Striatal cells (1.8 × 10^5^ per well) from 14-day embryonic Swiss mice (Janvier) were cultured in B27-supplemented Neurobasal medium (Invitrogen; 500 nM L-glutamine, 60 μg ml^−1^ penicillin G and 25 μM β-mercaptoethanol) on 35-mm dishes pre-coated with 50 μg ml^−1^ poly-D-lysine (Sigma) at 37 °C in humidified 95% air and 5% CO_2_. After 7 days in culture, neurons were transfected with WT DARPP-32-GFP or DARPP-32 Ser97 mutants, S97A or S97D (1 μg), as well as myc-β-adducin (1 μg) DNA, using Lipofectamine LTX in OptiMEM serum-free medium (Invitrogen). For acceptor photobleaching neurons were co-transfected with GFP or DARPP-32-GFP as above and mCherry or an mCherry–β-adducin fusion protein generated by subcloning β-adducin into the pmcherry-N1 vector (Clontech, #632523) using the Phusion High-Fidelity PCR kit (New England Biolabs, #M0531S).

### Fluorescence recovery after photobleaching

For FRAP experiments, cells were placed in HBSS medium at 32 °C on a Leica SP5 II upright microscope (Leica Microsystems). Images were acquired with a × 40 HCX APO (0.80 NA (numerical aperture)) water immersion objective and the FRAP experiment was performed with the FRAP Wizard software from Leica Microsystems. Ten images where taken at low laser intensity (∼5%) before the bleach for measuring basal fluorescence intensity. Photobleaching was done at 100% of the 488-nm laser line during 500 ms. Recovery was followed with the same laser power as in the pre-bleached session at the same rate of imaging for 40 s. For each time point, the intensity of the bleached area was normalized to the pre-bleached intensity. FRAP recovery curves and analysis were generated using Matlab (Matworks).

### Acceptor photobleaching

Fixed cells were imaged using a Leica TCS SP5 upright confocal microscope using the FRET acceptor photobleaching wizard and a × 40 0.8 NA water immersion objective (Leica Microsystems). Pre-bleach and post-bleach images were serially recorded by excitation of GFP at 488 nm (donor channel) with an argon laser and mCherry at 561 nm (acceptor channel). Low laser intensities were used to avoid bleaching effects during acquisition. Neurons were selected by visualizing only the donor channel to prevent premature partial bleaching of the acceptor. The acceptor was bleached with high intensity (100%) power at the 543-nm laser line for two iterations. Images were analysed using Matlab (Mathworks). The change in the fluorescence intensity between pre- and post-bleach donor values (efficiency, E) was calculated using the formula *E*=(donor after/donor before) × 100/donor after, and was shown as a percentage; pseudocoloured images showing FRET efficiency values were also generated.

### Immunohistochemistry of mouse brain sections

Brains from mice perfused for 5 min with 4% paraformaldehyde, 50 mM NaF were postfixed at 4 °C O/N and cut on a vibratome (Leica) into 30-μm sections (Bregma 1.3–0.6 mm). Free-floating sections were incubated overnight with antibodies at 4 °C, and after rinses in TBS, for 2 h at room temperature with secondary antibody (1:400 dilution; Cy3-coupled anti-rabbit IgG or Alexa488-conjugated anti-mouse antibodies; Molecular Probes). Nuclei were labelled with DAPI-containing vectashield (Vector) and photographed at × 63 magnification with a Leica-SP5-confocal microscope. At least four images were taken per brain area and animal. Analysis was performed using ImageJ, in which the percentage of cells with predominantly nuclear DARPP-32 was measured. For synaptic clusters analysis antibodies for β-adducin (1:500; as above) and PSD-95 (1:300; Upstate Biotechnology) and secondary antibodies (Alexa Fluor 488 and 555, 1:250, Jackson ImmunoResearch) were used. F-actin was labelled with phalloidin–rhodamine (1:1,000; Sigma). Stained coronal sections were examined blinded to genotype by confocal microscopy, using a Leica SP5 laser scanning confocal spectral microscope with argon and helium–neon lasers. Images were taken with a × 63 numerical aperture lens with × 4 digital zoom and standard (1 Airy disc) pinhole. For each mouse (*n*=4), at least three slices of 30 μm containing striatal tissue were analysed. Up to three representative images, from ventral striatum, were obtained from each slice. For each image, the entire three-dimensional stack of images was obtained by the use of the Z drive present in the Leica SP5 microscope, and the size of the optical image was 0.5 μm, with a separation of 2 μm between each. The number of double-labelled β-adducin/PSD-95- or β-adducin/F-actin-positive clusters was counted by the freeware NIH ImageJ (Wayne Rasband, NIH).

### Golgi-Cox staining, analysis of spine density and morphology

Golgi staining was performed according to the published protocols^59^,^60^. Essentially, mouse brain hemispheres were incubated in the dark for 14–17 days in a dye consisting of 1% potassium dichromate, 1% mercury chloride and 0.8% potassium chromate (all w/v, filtered). The tissue was then washed 3 × 2 min in aqua dest. and 30 min in 90% EtOH (v/v). Two hundred-μm sections were cut in 70% EtOH on a vibratome (Leica) and washed in aqua dest. for 5 min. Next, they were reduced in 16% ammonia solution for 1 h before washing in aqua dest. for 2 min and fixation in 1% sodium thiosulfate (w/v) for 7 min. After a final wash in aqua dest. for 2 min, sections were mounted on superfrost coverslips. For dehydration, they were left for 3 min in 50%, then 70, 80 and 100% EtOH. Incubation for 2 × 5 min in a 2:1 isopropanol:EtOH mixture was followed by 1 × 5 min in pure isopropanol and 2 × 5 min in xylol. Finally, samples were mounted with mounting medium (Eukitt) and left for 24 h to settle. Secondary dendrites from nucleus accumbens were photographed, with a maximum of two dendrites per neuron and from at least two slices per animal. Z-stacks from 0.2-μm sections were obtained in bright field at × 100 resolution on a DM6000-2 microscope (Leica) (Supplementary Fig. 8a). Files were analysed with the ImageJ software (Supplementary Fig. 8b). First, Z-stacks were summed using the plugin Bio-format importer. The scale was adjusted according to the pixel size of the images. To improve image quality, images were deconvoluted and brightness/contrast adjusted. The total number of spines was obtained using the cell counter tool. At least 40 dendrites per group from at least three mice per group were counted. To analyse spine subtypes, at least five dendrites per mouse were analysed. On average it was aimed to analyse the first 20 spines on the dendrites that were clearly observed in the X–Y plane. Between 315 and 345 spines per group were analysed for major head diameter as well as neck length. Spines were analysed as follows (Supplementary Fig. 8c). Each spine was categorized as having or not having a neck. Spines were defined as stubby if they did not contain a visible neck. Spines with necks were separated into thin and mushroom spines based on head width. Filopodia, defined as protrusions >1.5 μm in length without a neck, were excluded from the analysis of spine subtypes. Spines with heads less than the average width were categorized as thin, and those with heads greater than the average width were categorized as mushroom, as described^61^. From these measures, the percentage of the various spine types were obtained. On the basis of the total values of spine number calculated above, the percentage was converted into absolute numbers. Spine length was measured as the sum of neck length and minor head diameter and spine width was measured as the major head diameter (Supplementary Fig. 8c). Picture acquisition and subsequent analysis were performed independently by two investigators and results were then pooled. Overall differences between the results were minor.

### Punch, immunoblot and actin analyses

Immediately after killing the mice, heads were frozen for 8 s in liquid nitrogen. Skin and bones were removed on dry ice and the brains were cut with a cryostat (Leica) into 200-μm sections. The striatum and nucleus accumbens were removed with small metal punches on dry ice. A volume of 300 μl of 1% SDS (w/v) were added and the samples were sonicated immediately for 10 s before placing them at 98 °C for 5 min. Laemmli buffer was added (see above) and the samples were heated for another 5 min. Samples were analysed using Bio-rad Mini Protean TGX-gels and the Bio-rad transfer system. Immunoblotting was performed either with a single primary antibody or sequentially in two steps: incubation with the main antibody of interest on the first day followed the next day by a second incubation with actin antibody (sometimes mixed with an antibody, which was not quantified but used as transfection, for example, DARPP-32), without prior stripping. For quantification of phospho and total proteins two different gels and blots were run for the same samples. Secondary antibody binding was detected by Odyssey infrared imaging apparatus (Li-Cor Inc., Lincoln, NE) and analysed using ImageJ. Original images of immunoblots shown in the figures and supplementary figures are shown in Supplementary Fig. 9. Alterations in the G-actin/F-actin ratio were determined using an *in vivo* assay kit (Cytoskeleton Inc., Denver, CO), essentially according to the manufacturer’s instructions. Briefly, tissues were lysed by using 27G syringes in a buffer that solubilizes G-actin but renders F-actin insoluble. Following high-speed centrifugation (100,000*g* at 37 °C for 1 h), F-actin was recovered in the pellet, whereas G-actin remained in the supernatant. The pellet was resuspended in F-actin depolymerization buffer and incubated on ice for 1 h to allow actin depolymerization to occur. Both fractions had the same volume to allow comparison. Then, samples were resolved by SDS–PAGE, electrotransferred to nitrocellulose and probed with anti-actin antibody provided with the kit. Total actin per mouse (used as a loading control) was determined by plotting the OD values from G- and F-actin bands. F-actin ratio was determined by calculating the percentage of F-actin OD band with respect to total actin.

### Behavioural analysis

For NEE, mice were housed (4–5 mice per cage) in a clean rat cage (40 × 26 × 17 cm) that contained fresh saw dust; at least two housing opportunities consisting of perforated plastic balls or igloos obtained from a pet shop; and two bent or straight plastic tubes, of which one was attached to the grid of the cage as well as various small plastic children’s toys. Locomotor activity after cocaine injection was assessed in a circular corridor (Imetronic)^62^. After a 30-min habituation phase to the apparatus, mice were injected with cocaine and locomotor activity was tracked for 60 min by measuring locomotion and rearings in time bins of 5 min.

### Statistical analysis

Sample sizes were chosen according to variability estimates in previous similar experiments or in pilot experiments. Data are expressed as means±s.e.m. Statistical analysis was performed in GraphPrism. The tests used were two-tailed Student’s *t*-test for comparison of two groups, one-way analysis of variance (ANOVA) when there were more than two groups and two-way ANOVA when two factors were varied. When ANOVA was significant, *post hoc* tests were Tukey’s test for one way and Šidák’s test for two way. For cumulative dendritic spine length and width analysis Gehan–Breslow–Wilcoxon test was used. The threshold for significance was *P*<0.05.

## Additional information

**How to cite this article:** Engmann, O. *et al.* DARPP-32 interaction with adducin may mediate rapid environmental effects on striatal neurons. *Nat. Commun.* 6:10099 doi: 10.1038/ncomms10099 (2015).

## Supplementary Material

Supplementary Information Supplementary Figures 1-9
