# Supplementary material for: DARPP-32 interaction with adducin may mediate rapid environmental effects on striatal neurons
Source: Nat Commun. 2015 Dec 7;6:10099. doi: 10.1038/ncomms10099 (PMC4675091; doi:10.1038/ncomms10099)
Supplement: Supplementary Information — Supplementary Figures 1-9 [file ncomms10099-s1.pdf]

Alpha adducin rat

1 MNGDTRAAVV TSPPPTTAPH KERYFDR**VDE NNPEYLR**ER**N MAPDLRQDFN**
51 **MMEQK**KRVSM ILQSPAFCEE LESMIQEQFK KGKNPTGLLA LQQIADFMTA
101 SVPNVYPAAP QGGMAALNMS LGMVTPVNDL R**GSDSIAYDK** GEKLLRCKLA
151 AFYRLADLFG WSQLIYNHIT TRVNSEQEHF LIVPFGLLYS EVTASSLVK**V**
201 **NLQGDIVDR**G STNLGVNQAG FTLHSAIYAA RPDAKCIVHI HTPAGAAVSA
251 MKCGLLPISP EALSLGEVAY HDYHGILVDE EEKILIQKNL GPKSKVLILR
301 NHGLVSVGES VEEAFFYIHN LVVACEIQVR **TLASAGGPDN LVLLDPGK**YK
351 AKSR**SPGTPA GEGSGSPPKW QIGEQEFEAL MRMLDNLGYR** TGYPYRYPAL
401 RERSKKYSDV EVPASVTGHS FASDGDSGTC SPLRHSFQKQ QREKTRWLNS
451 GRGDDASEEG QNGSSPKSKT K**WTKEDGHRT STSAVPNLFV PLNTNPK**EVQ
501 EMRNKIR**EQN LQDIK**TAGPQ SQVLCGVMMD R**SLVQGELVT ASK**AIIEK**EY**
551 **QPHVIVSTTG PNPFNTLTDR ELEEYR**REVE RKQK**GSEENL DETR**EQKEKS
601 PPDQSAVPNT PPSTPVK**LEG GLPQEPTSR**D GSGCHASKPT PPDLSPDEPS
651 EALAFPTVAE EAHASPDTTQ PLAEADPEPA SASAPGAEEV ASPATEEGSP
701 MDPGSDGSPG KSPSKKKKKF RTPSFLKKSK KKSDS

Beta-adducin isoform a rat

1 MSEDTVPEAA SPPPSQGQHY FDR**FSEDDPE YLR**LRNRAAD LR**QDFNLMEQ**
51 **K**KR**VTMILQS PSFREELEGL IQEQMKKGNN SSNIWALR**QI ADFMASTSHA
101 VFPASSMNFS MMTPINDLHT ADSLNLAKGE RLMRCKISSV YRLLDLYGWA
151 QLSDTYVTLR VSKEQDHFLI SPKGVSCSEV TASSLIK**VNI LGEVVEK**GSS
201 CFPVDTTGFS LHSAIYAARP DVRCAIHLHT PATAAVSAMK CGLLPVSHNA
251 LLVGDMAYYD FNGEMEQEAD RINLQKCLGP TCKILVLRNH GMVALGDTVE
301 EAFYKVFHLQ AACEVQVSAL SSAGGTENLI LLEQEKHRPH EVGSVQWAGS
351 TFGPMQKSRL GEHEFEALMR **MLDNLGYR**TG YTYRHPFVQE KTKHK**SEVEI**
401 **PATVTAFVFE EDGVPVPALR** QHAQKQQKEK TR**WLNTPNTY LRVNVADEVQ**
451 **R**NMGSPRPKT TWMKADEVEK **SSSGMPIRIE NPNQFVPLYT DPQEVLDMR**N
501 KIREQNRQDI K**SAGPQSQLL ASVIAEK**SR**S PSTESQLASK** GDADTKDELE
551 ETVPNPFSQL TDQELEEYKK EVERKKLEQE QEGEKDAATE EPGSPVK**STP**
601 **ASPVQSPTR**A GTKSPAVSPS KASEDAKKTE VSEANTEPEP EKPEGVVVNG
651 K**EEEPSVEEV LSKGPGQMTT NADTDGDSYK** DK**TESVTSGP LSPEGSPSK**S
701 PSKKKKKFRT PSFLKKSKKK EKVES

Supplementary Fig. 1. Mass spectrometry identification of adducin peptides in DARPP-32 binding proteins. Lysates from rat striatum were incubated with DARPP-32-Sepharose or casein-Sepharose. After washing, bound proteins were eluted with a NaCl step gradient. Major bands apparent on Coomassie-stained polyacrylamide gel in DARPP-32 eluates but not casein eluates were excised. After tryptic digestion peptides were identified by LC-MS/MS. The analysis of a band migrating with an apparent MW of 120,000 and eluted from DARPP-32 identified α-adducin (gi|785039, 26.5% coverage, $p = 2.4\ 10^{-51}$) and β-adducin isoform a (gi|158081763, 31.7% coverage, $p = 3.4\ 10^{-65}$). Peptides from these two proteins were not recovered from casein-Sepharose. The identified peptides are indicated in bold, highlighted yellow.

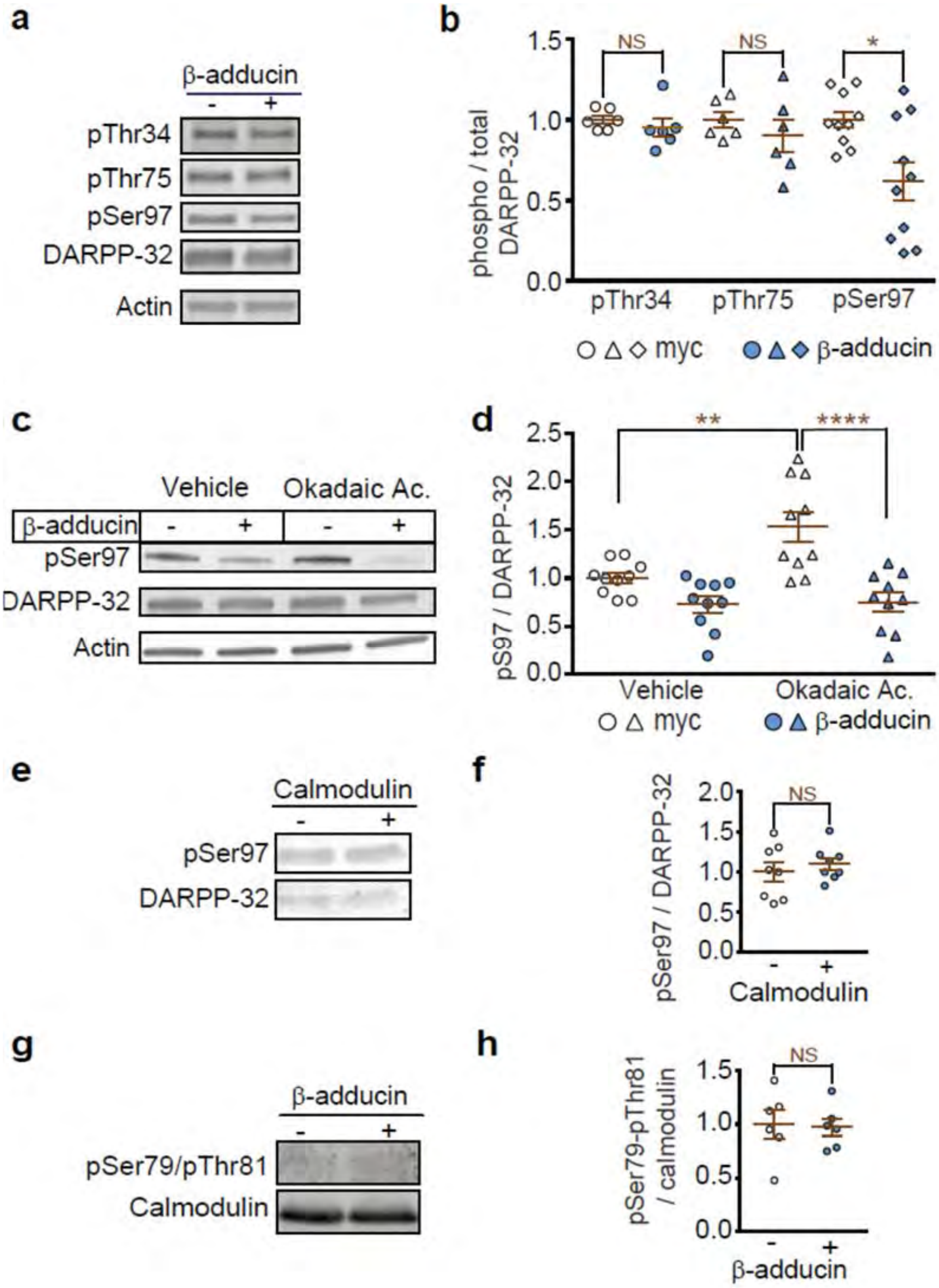

Supplementary Fig. 2. β-adducin alters phosphorylation of DARPP-32 Ser97. a) COS7 cells were co-transfected with DARPP-32-GFP and myc vector (-) or myc-β-adducin (+).pThr34, pThr75, and pSer97 in DARPP-32, the total amounts of this protein, and actin as loading control, were analyzed by immunoblotting of COS-7 cells lysates. **b)** Quantification of results as in (a). Data are means + SEM; n = 6-10 per group from >3 experiments; Student's *t*-test, pThr34, t_{10} = 0.78, NS, pThr75, t_{10} = 0.89, NS, pSer97, t_{18} = 2.92, p<0.01. **c)** COS7-cells were transfected with DARPP-32-GFP in the presence of absence of myc-β-adducin and incubated for 30 min with vehicle (-) or okadaic acid (200 nM, +). pSer97 and total DARPP-32, and actin as loading control, were analyzed by immunoblotting. **d)** Quantification

of results as in (c). Data are means + SEM; n=10 per group from 3 experiments; two-way ANOVA: β-adducin factor, $F_{(1,36)} = 25.89$, $p<10^{-4}$; okadaic acid factor, $F_{(1,36)} = 7.17$, $p = 0.01$; interaction, $F_{(1,36)} = 6.09$, $p = 0.02$; Šidák post-hoc test, **, $p<0.01$, ****, $p<10^{-4}$. **e)** Purified DARPP-32 was incubated with CK2 and 100 µM ATP in the absence (-) or presence (+) of calmodulin. Phosphorylation of Ser97 (pSer97) and total DARPP-32 were analyzed by immunoblotting. **f)** Quantification of results as in (e). Data are means + SEM; n=8 per group from 2 experiments; Student's *t*-test: $t_{14} = 0.72$, NS. **g)** Conditions were as in (c) except that DARPP-32 was replaced by calmodulin as CK2 substrate. Calmodulin phosphorylation on Ser79/Thr81 (pSer79/pThr81) and total calmodulin were analyzed by immunoblotting. **h)** Quantification of results as in (g). Data are means + SEM; n = 6 per group from 2 experiments; Student's *t*-test, $t_{10} = 0.18$, NS.

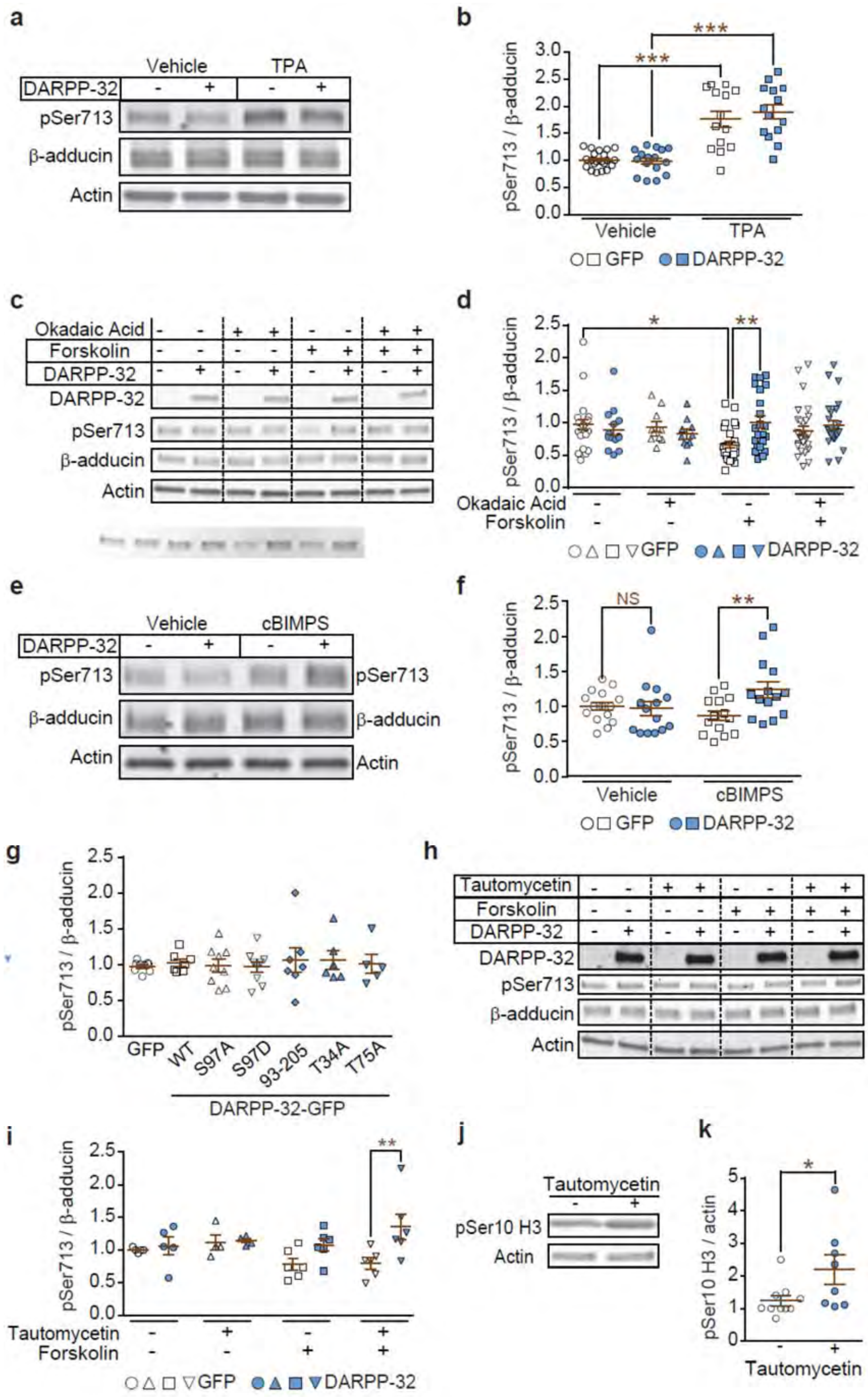
a
Vehicle
TPA
DARPP-32
pSer713
β-adducin
Actin
b
pSer713 / β-adducin
Vehicle
TPA
GFP
DARPP-32
c
Okadaic Acid
Forskolin
DARPP-32
pSer713
β-adducin
Actin
d
pSer713 / β-adducin
Okadaic Acid
Forskolin
GFP
DARPP-32
e
Vehicle
cBIMPS
DARPP-32
pSer713
β-adducin
Actin
f
NS
pSer713 / β-adducin
Vehicle
cBIMPS
GFP
DARPP-32
g
pSer713 / β-adducin
GFP
WT
S97A
S97D
93-205
T34A
T75A
DARPP-32-GFP
h
Tautomycetin
Forskolin
DARPP-32
pSer713
β-adducin
Actin
i
pSer713 / β-adducin
Tautomycetin
Forskolin
GFP
DARPP-32
j
Tautomycetin
pSer10 H3
Actin
k
pSer10 H3 / actin
Tautomycetin

Supplementary Fig. 3. DARPP-32 modulates cAMP-induced phosphorylation of β-adducin Ser713. a) COS7 cells co-transfected with myc-β-adducin and GFP (DARPP-32 -) or DARPP-32-GFP (DARPP-32 +) were incubated for 20 min with vehicle (DMSO, 1:1,000 final) or 50 nM TPA. Samples were analyzed by immunoblotting with the indicated antibodies. **b)** Quantification of results as in (a). Data are means + SEM; n=14-21 per group; two-way ANOVA: TPA factor: $F_{(1,61)} = 79.8$, $p<0.\ 10^{-4}$; DARPP-32 factor, $F_{(1,61)} = 0.31$, NS; no interaction, $F_{(1,61)} = 0.61$, NS; Šidák post-hoc test vs vehicle, ***, $p<0.001$. **c)** COS7-cells were co-transfected with myc-β-adducin and either GFP (DARPP-32 -) or DARPP-32-GFP (DARPP-32 +) and incubated for 30 min in the absence (-) or presence (+) of forskolin (100 µM) and okadaic acid (OA, 200 nM), as indicated. pSer713, total β-adducin, DARPP-32, and actin as loading control, were analyzed by immunoblotting. **d)** Quantification of results as in (c). Data are means + SEM; n=10-29 per group from >3 experiments; two-way ANOVA: drugs factor, $F_{(3,153)} = 0.4827$, $p = 0.69$; DARPP-32 factor, $F_{(1,153)} = 1.05$, $p = 0.3$; interaction $F_{(3,153)} = 3.10$, $p = 0.03$; Šidák post-hoc test, *, $p<0.05$, **, $p<0.01$. **e)** COS7 cells were co-transfected as in (a) and incubated for 20 min with vehicle (DMSO 1/1,000 final) or the cell permeant cAMP-analog Sp 5,6-DCl-cBIMPS (cBIMPS, 10 µM). Samples were analyzed by imunoblotting as in (a). **f)** Quantification of results as in (e). Data are means + SEM. N = 16-25 per group, >3 experiments; two-way ANOVA: cBIMPS factor, $F_{(1,62)} = 0.01$, NS; DARPP-32 factor, $F_{(1,62)} = 5.77$, $p = 0.02$; interaction, $F_{(1,77)} = 4.78$, $p = 0.03$; Šidák post-hoc test vs vehicle, **, $p<0.01$. **g)** COS7-cells were transfected with myc-β-adducin and GFP or various forms of DARPP-32-GFP, as indicated. Cells were incubated for 20 min with 1:1,000 DMSO (vehicle) and lysates were analyzed by immunoblotting for pSer713 and β-adducin. Data are means + SEM; n = 5-9 per group; one-way ANOVA: $F_{(6,45)} = 1.2$, $p = 0.32$. **h)** COS7-cells were co-transfected with myc-β-adducin and either GFP (DARPP-32 -) or DARPP-32-GFP (DARPP-32 +) and incubated for 30 min with tautomycetin (200 nM), forskolin (100 µM), or both, as indicated. DARPP-32, pSer713 and total β-adducin, and actin as loading control, were analyzed by immunoblotting. **i)** Quantification of results as in (h). Data are means + SEM; n=4-6 per group from 3 experiments; two-way ANOVA: DARPP-32 factor, $F_{(3,34)} = 7.77$, $p = 0.009$; drugs factor, $F_{(3,34)} = 1.00$, $p = 0.4$; interaction $F_{(3,34)} = 2.12$, $p = 0.12$; Šidák post-hoc test, **, $p<0.01$. **j)** COS7-cells were treated for 30 min with 50 µM tautomycetin and lysates were immunoblotted for pSer10 histone H3 and actin as a loading control. **k)** Quantification of results as in (j). Data are means + SEM; n = 8-10 per group; Student's *t*-test, $t_{(16)} = 2.23$, *, $p<0.05$.

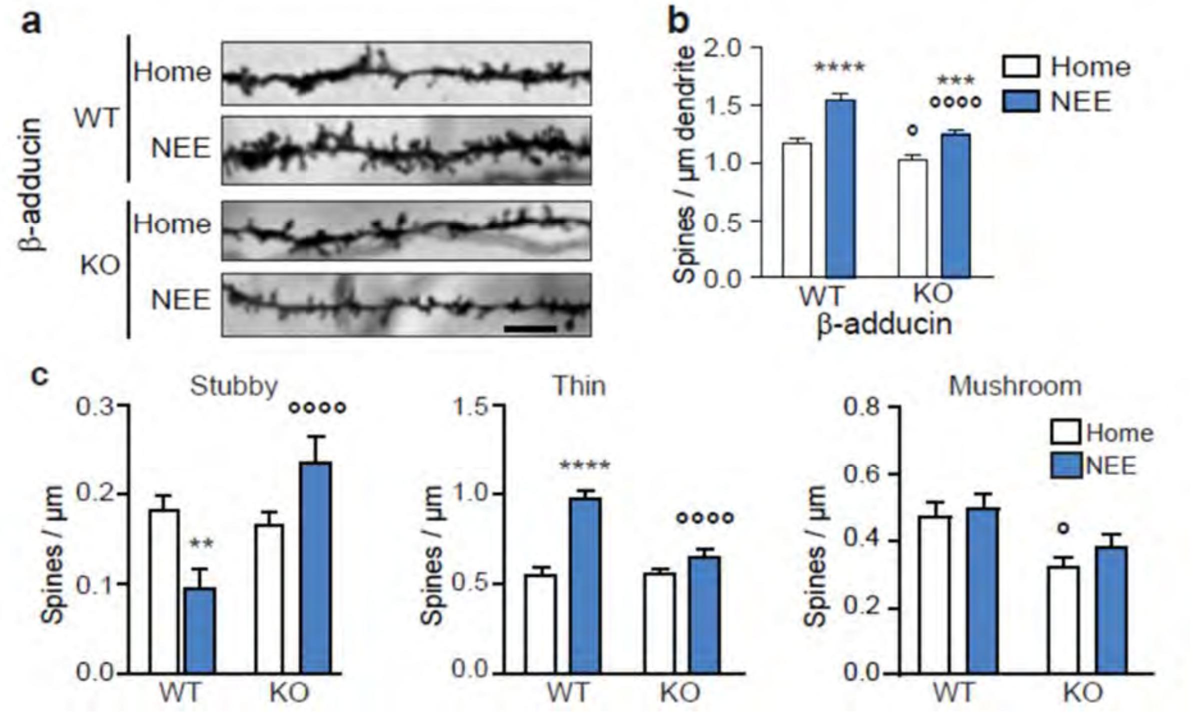

Supplementary Fig. 4. Role of β-adducin in dendritic spines alterations induced by NEE. (a) Wild-type and β-adducin KO littermate mice were housed in their home cage or exposed to 24-h NEE before sacrifice. Dendritic spines were visualized by Golgi-cox stain. Scale bar: 2 µm. **b)** Spine density was measured with ImageJ in 45-59 dendrites per group of 3 animals. Two-way ANOVA: housing factor $F_{(1,211)} = 46.75$, $p<10^{-4}$; genotype effect $F_{(1,211)} = 25.92$, $p<10^{-4}$; no interaction $F_{(1,211)}=2.97$. Šidák post-hoc test, NEE vs home cage, ***, p<0.001, ****, $p<10^{-4}$; KO vs wild-type, °, p<0.05, °°°°, $p<10^{-4}$. **c)** On a subset of dendrites (16-20 dendrites per group) spines were classified according to their morphology and the density of stubby, thin, and mushroom spines was measured separately. Data are means + SEM; two-way ANOVA: stubby spines, housing effect, $F_{(1,74)} = 0.13$, p = 0.7, NS; genotype effect, $F_{(1,74)} = 8.64$, p=0.004; interaction, $F_{(1,74)} = 13.32$, p=0.0005; thin spines, housing effect $F_{(1,74)} = 33.31$, $p<10^{-4}$; genotype effect $F_{(1,74)} = 12.83$, p=0.0006; interaction $F_{(1,74)} = 14.06$, p=0.0003; mushroom spines, housing effect $F_{(1,74)} = 1.13$, p = 0.29, NS; genotype effect: $F_{(1,74)} = 10.94$, p=0.0015; no interaction $F_{(1,74)} = 0.18$; Šidák post-hoc test, NEE vs home cage, **, p<0.01, ****, $p<10^{-4}$; KO vs WT, °, p<0.05, °°°°, $p<10^{-4}$.

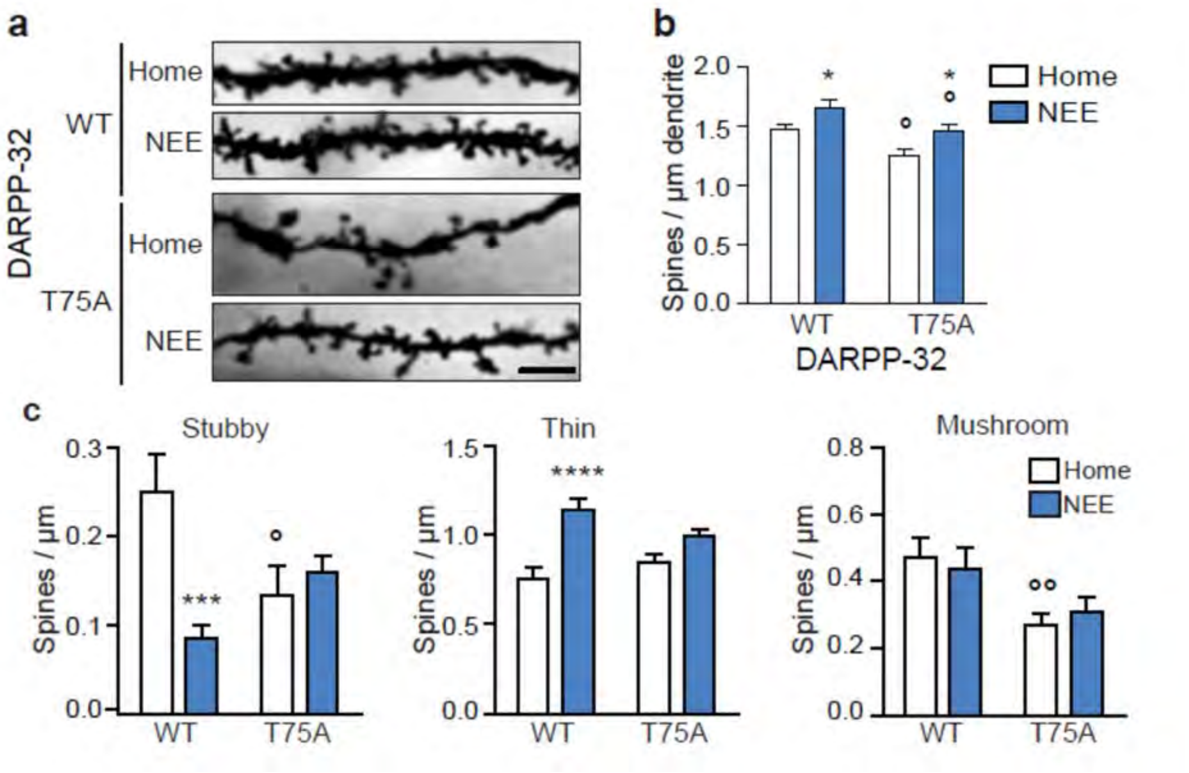

Supplementary Fig. 5. Role of DARPP-32 Thr34 in dendritic spines alterations induced by NEE. (a) Wild-type and T75A DARPP-32 mutant littermate mice were housed in their home cage or exposed to 24-h NEE before sacrifice and dendritic spines were visualized by Golgi-cox stain as in (Suppl. Fig. 4a). Scale bar: 2 µm. **b)** Spine density was measured in 42-52 dendrites per group of 3 animals. Two-way ANOVA: housing factor $F_{(1,193)} = 12.32$, $p<0.001$; genotype effect $F_{(1,193)} = 14.47$, $p<0.001$; no interaction $F_{(1,193)}=0.005$. Šidák post-hoc test, NEE vs home cage, *, $p<0.05$; T75A vs wild-type, °, $p<0.05$. **c)** On a subset of dendrites (16-20 dendrites per group) spines were classified according to their morphology and the density of stubby, thin, and mushroom spines was measured separately. Data are means + SEM; two-way ANOVA: stubby spines, housing effect, $F_{(1,67)} = 5.12$, $p<0.05$; genotype effect, $F_{(1,67)} = 0.45$, $p = 0.5$, NS; interaction, $F_{(1,67)} = 9.56$, $p<0.01$; thin spines, housing effect $F_{(1,67)} = 25.31$, $p<10^{-4}$; genotype effect $F_{(1,67)} = 0.28$, $p = 0.6$, NS; interaction $F_{(1,67)} = 5.10$, $p<0.05$; mushroom spines, housing effect $F_{(1,67)} = 0.003$, $p = 0.96$, NS; genotype effect: $F_{(1,67)} = 10.67$, $p<0.01$; no interaction $F_{(1,67)} = 0.50$, NS; Šidák post-hoc test, NEE vs home cage, *, $p<0.05$, ***, $p<0.001$, ****, $p<10^{-4}$; T75A vs WT, °, $p<0.05$, °°, $p<0.01$.

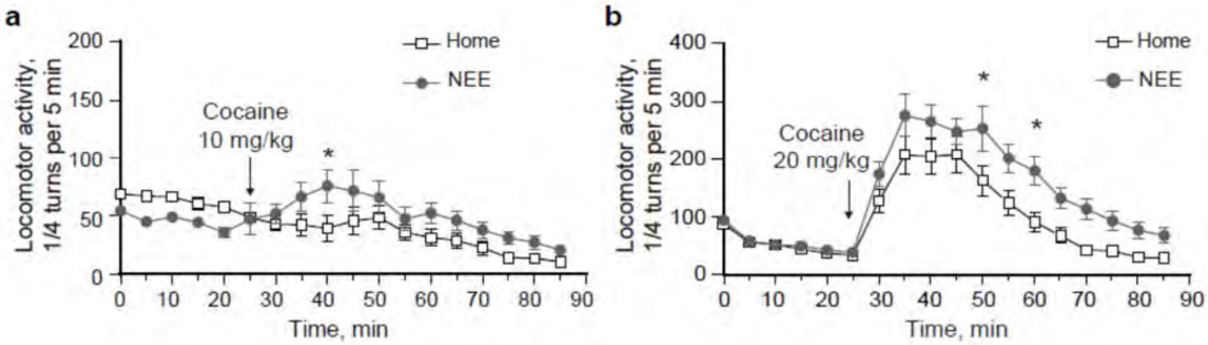

Supplementary Fig. 6. Effects of exposure to NEE on cocaine-induced locomotor activity. a) C57BL/6 mice were housed in their home cage or in a novel enriched environment (NEE) for 24 h. After a 30-min habituation in a circular maze, all mice were injected with 10 mg/kg cocaine i.p. (arrow). Locomotor activity was recorded in 5-min bins; 16 mice per group in 2 experiments. Two-way ANOVA: housing factor, $F_{(1,540)} = 6.625$, $p = 0.01$; time factor, $F_{(17,540)} = 6.213$, $p<10^{-4}$; interaction $F_{(1,540)} = 2.457$; Šidák post-hoc test for each time bin, *, $p<0.05$. **b)** Same as in (a) except that different batches of mice (14-15 mice per group in 3 experiments) were injected with 20 mg/kg cocaine i.p. (arrow). Two-way ANOVA: housing factor, $F_{(1,486)} = 46.03$, $p<10^{-4}$; time factor, $F_{(17,486)} = 30.61$, $p<10^{-4}$; no interaction $F_{(1,486)}=1.42$; Šidák post-hoc test for each time bin, *, $p<0.05$.

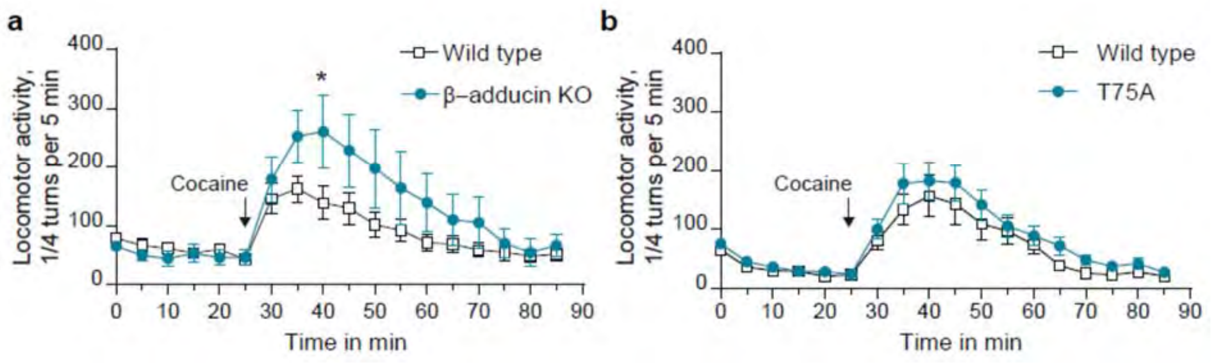

Supplementary Fig. 7. Effects of β-adducin KO and T75A DARPP-32 mutation on cocaine-induced locomotor activity. Data in this figure are identical to those in Fig. 9 for home cage-housed mice and are shown to allow direct visualization of the mutation effects. **a)** Wild type (n = 15) and β-adducin KO (n = 8) littermate mice were housed in their home cage; after a 30-min habituation in a circular maze, all mice were injected with 20 mg/kg cocaine i.p. (arrow). Locomotor activity was recorded in 5-min bins. Two-way ANOVA: genotype factor, $F_{(1,378)} = 17.49$, $p<10^{-4}$; time factor, $F_{(17,378)} = 9.42$, $p<10^{-4}$; interaction, $F_{(17,378)} = 1.56$, NS. Šidák post-hoc test for each time bin, *, p<0.05. a, b) Data are means +/- SEM. **b)** Wild type (n = 10) and T75A DARPP-32 (n = 13) littermate mice were treated and studied as in (a). Two-way ANOVA: genotype factor, $F_{(1,378)} = 8.65$, p = 0.003; time factor, $F_{(17,378)} = 17,18$, $p<10^{-4}$; interaction, $F_{(17,378)} = 0.26$, NS. Šidák post-hoc test for each time bin, no single point was significantly different between genotypes. a, b) Data are means +/- SEM.

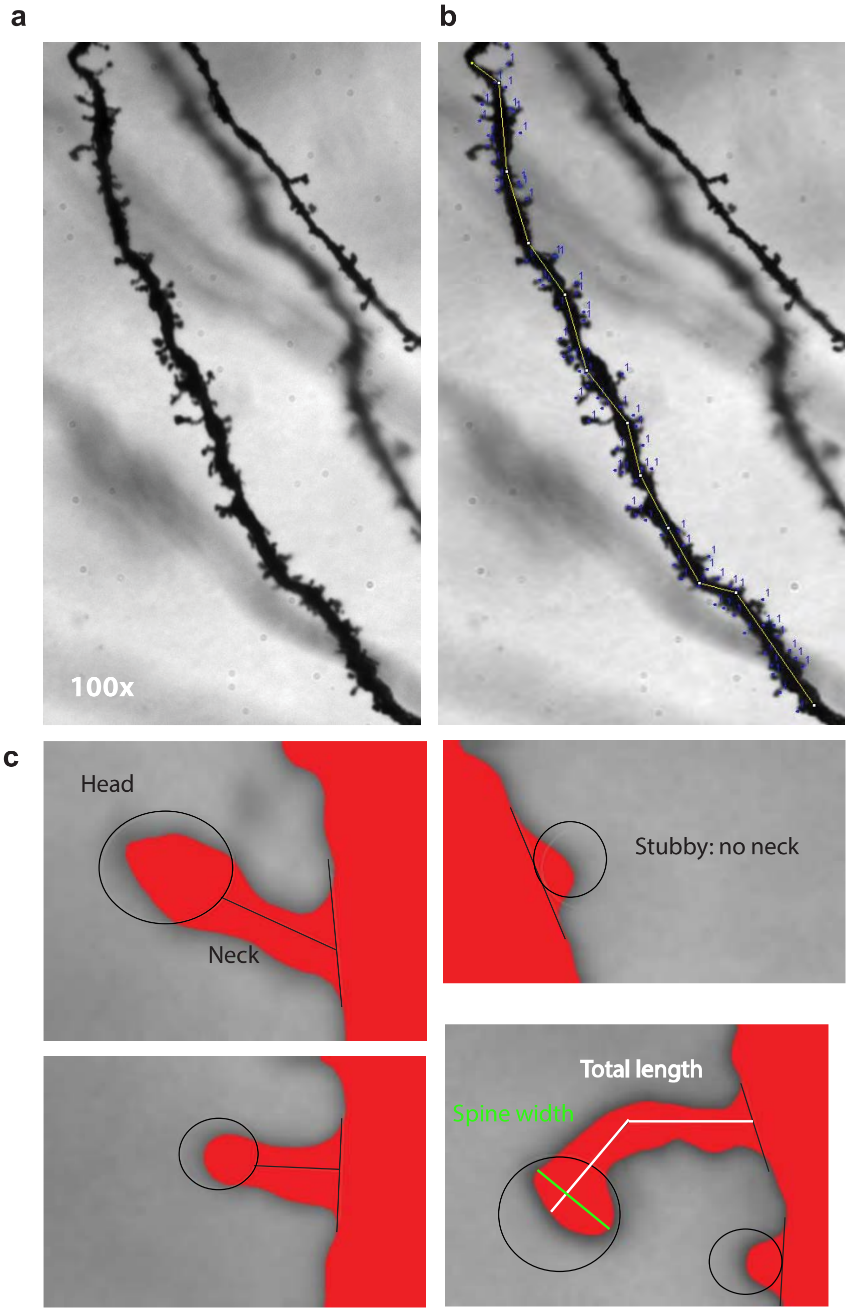

Supplementary Fig. 8. Methods for spine measurement. a) Nucleus accumbens sections were stained with the Golgi-cox method. Z-stack of 0.2 µm optical sections were acquired in bright field at 100x resolution on a DM6000-2 microscope (Leica). **b)** Analysis of spines using ImageJ software. Spines were counted using the cell counter tool. **c)** Spines were categorized as having or not having a neck. Spines were defined as stubby if they did not contain a visible neck (right, top). Spines with necks were separated into mushroom and thin spines based on head width. Spines with heads greater than the average width were categorized as mushroom (left, top), and those with heads less than the average width were categorized as thin (left, bottom). Spine length was measured as the sum of neck length and minor head diameter (right, bottom). Spine width was measured as the major head diameter.

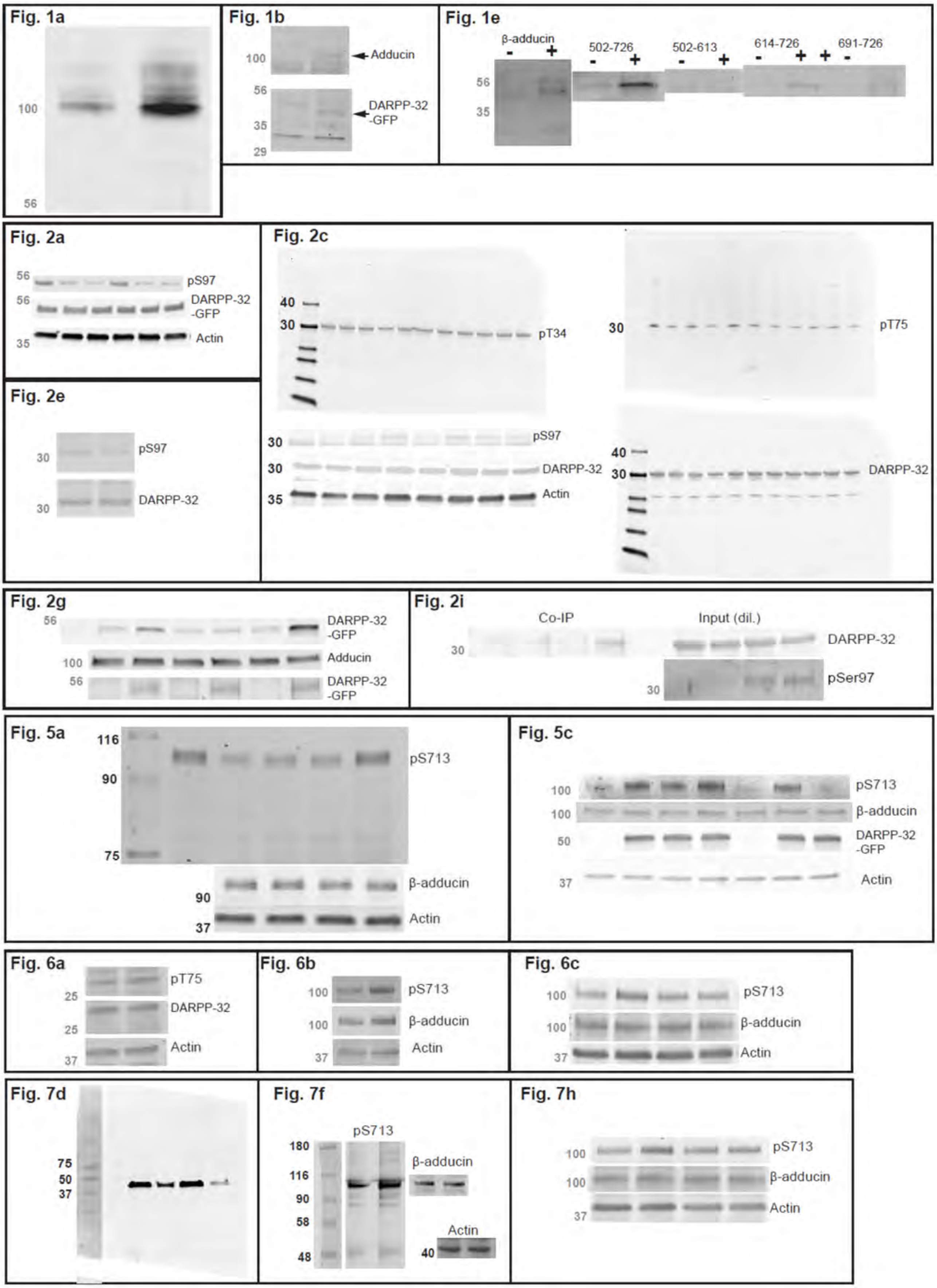

Fig. 1a
100
56
Fig. 1b
100
Adducin
56
35
29
DARPP-32
-GFP
Fig. 1e
β-adducin
- +
502-726
- +
502-613
- +
614-726
- +
691-726
+ -
56
35
Fig. 2a
56
pS97
56
DARPP-32
-GFP
35
Actin
Fig. 2c
40
30
pT34
30
pT75
30
pS97
30
DARPP-32
35
Actin
40
30
DARPP-32
Fig. 2e
30
pS97
30
DARPP-32
Fig. 2g
56
DARPP-32
-GFP
100
Adducin
56
DARPP-32
-GFP
Fig. 2i
Co-IP
Input (dil.)
30
DARPP-32
30
pSer97
Fig. 5a
116
90
75
pS713
β-adducin
90
37
Actin
Fig. 5c
100
pS713
100
β-adducin
50
DARPP-32
-GFP
37
Actin
Fig. 6a
pT75
25
DARPP-32
25
Actin
37
Fig. 6b
100
pS713
100
β-adducin
37
Actin
Fig. 6c
100
pS713
100
β-adducin
37
Actin
Fig. 7d
75
50
37
Fig. 7f
pS713
180
116
90
58
48
β-adducin
Actin
40
Fig. 7h
100
pS713
100
β-adducin
37
Actin

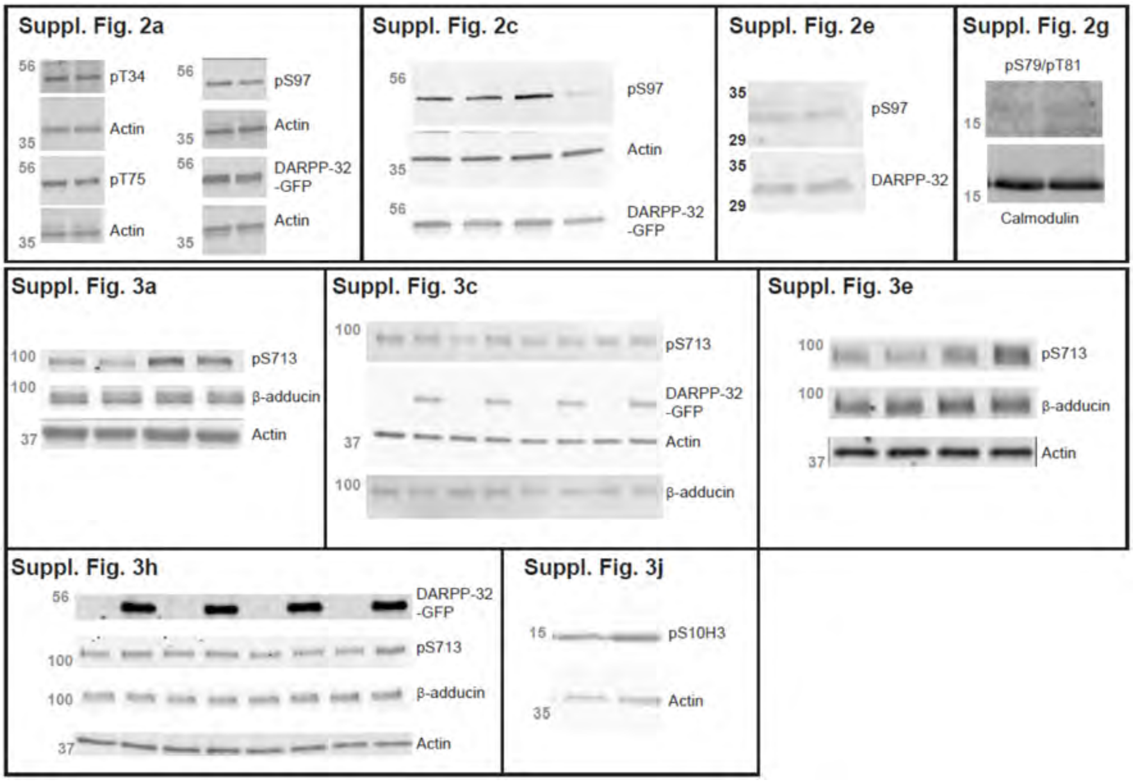

Supplementary Fig. 9. Immunoblots used in Figures and Supplementary Figures, as indicated. Immunoblots were revealed with infrared fluorescent antibodies detected with a LiCor Odyssey apparatus. Proteins of interest were identified using molecular weight markers or known proteins of reference. Molecular weight markers positions (kDa) are indicated in black (actual) or grey (estimated). In most experiments only cropped images including the proteins of interest were saved and are shown here. The antibody used or the position of proteins of interest are indicated.
